# Design of FPGA-Based SHE and SPWM Digital Switching Controllers for 21-Level Cascaded H-Bridge Multilevel Inverter Model

**DOI:** 10.3390/mi13020179

**Published:** 2022-01-25

**Authors:** Emilia Noorsal, Asyraf Rongi, Intan Rahayu Ibrahim, Rosheila Darus, Daniel Kho, Samsul Setumin

**Affiliations:** 1 School of Electrical Engineering, College of Engineering, Universiti Teknologi MARA, Cawangan Pulau Pinang, Kampus Permatang Pauh, Permatang Pauh 13500, Malaysia; asyraf.rongi@firstcity.edu.my (A.R.); intan121@uitm.edu.my (I.R.I.); roshe678@uitm.edu.my (R.D.); samsuls@uitm.edu.my (S.S.); 2 Faculty of Engineering and Computing, First City University College, No.1, Persiaran Bukit Utama, Bandar Utama, Petaling Jaya 47800, Malaysia; 3 LogikHaus Sdn. Bhd. (1314363-U), c/o Forward School, 2 Lebuh Achech, Georgetown 10450, Malaysia; daniel.kho@logik.haus

**Keywords:** multilevel inverter, selective harmonic elimination (SHE), sinusoidal pulse width modulation (SPWM), FPGA, hardware description language (HDL), Verilog codes, total harmonic distortion (THD)

## Abstract

Multilevel inverters are a type of power electronic circuit that converts direct current (DC) to alternating current (AC) for use in high-voltage and high-power applications. Many recent studies on multilevel inverters have used field-programmable gate arrays (FPGAs) as a switching controller device to overcome the limitations of microcontrollers or DSPs, such as limited sampling rate, low execution speed, and a limited number of IO pins. However, the design techniques of most existing FPGA-based switching controllers require large amounts of memory (RAM) for storage of sampled data points as well as complex controller architectures to generate the output gating pulses. Therefore, in this paper, we propose two types of FPGA-based digital switching controllers, namely selective harmonic elimination (SHE) and sinusoidal pulse width modulation (SPWM), for a 21-level multilevel inverter. Both switching controllers were designed with minimal hardware complexity and logic utilisation. The designed SHE switching controller mainly consists of a four-bit finite state machine (FSM) and a 13-bit counter, while the SPWM switching controller employs a simple iterative CORDIC algorithm with a small amount of data storage requirement, a six-bit up-down counter, and a few adders. Initially, both digital switching controllers (SHE and SPWM) were designed using the hardware description language (HDL) in Verilog codes and functionally verified using the developed testbenches. The designed digital switching controllers were then synthesised and downloaded to the Intel FPGA (DE2-115) board for real-time verification purposes. For system-level verification, both switching controllers were tested on five cascaded H-Bridge circuits for a 21-level multilevel inverter model using the HDL co-simulation method in MATLAB Simulink. From the synthesised logic gates, it was found that the designed SHE and SPWM switching controllers require only 186 and 369 logic elements (LEs), respectively, which is less than 1% of the total LEs in an FPGA (Cyclone IV E) chip. The execution speed of the SHE switching controller implemented in the FPGA (Cyclone IV E) chip was found to be a maximum of 99.97% faster when compared with the microcontroller (PIC16F877A). The THD percentage of the 21-level SHE digital switching controller (3.91%) was found to be 37% less than that of the SPWM digital switching controller (6.17%). In conclusion, the proposed simplified design architectures of SHE and SPWM digital switching controllers have been proven to not only require minimal logic resources, achieve high processing speeds, and function correctly when tested on a real-time FPGA board, but also generate the desired 21-level stepped sine-wave output voltage (±360 V_PP_) at a frequency of 50 Hz with low THD percentages when tested on a 21-level cascaded H-Bridge multilevel inverter model.

## 1. Introduction

Over the last decade, multilevel inverters (MLIs) have garnered significant research attention in high-voltage and high-power applications, such as high-power motor drives, power conditioning, renewable energy conversion, and power distribution, due to their simple structure, modularity, and transformer-less circuit [1,2,3,4]. The primary role of a multilevel inverter is to convert direct current (DC) input to alternating current (AC) output by generating a staircase of AC output waveforms with a low value of high-frequency distortion [4,5]. To compensate for the low supply voltage, the semiconductor power switches are coupled to several low-DC sources, which are then configured into multilevel structures to produce a high-power output. These power switches accomplish power conversion with the assistance of a digital switching controller by synthesising multiple DC voltage sources into a high-voltage stepped output waveform [3,6,7]. The advantages of a multilevel inverter over a two-level inverter include improved output-voltage quality, low switching losses, high-voltage capability, reduced total harmonic distortion, and reduced dv/dt stresses on semiconductor switches, all of which indirectly reduce electromagnetic compatibility (EMC) issues [3,4,6,7,8].

Several topological architectures of multilevel inverters have been adopted to meet essential requirements, such as low switching-device count, ability to withstand high-voltage signals, and a lower switching frequency for the switching devices [3,4,6]. Diode-clamped [9,10], flying-capacitor [11], and cascaded H-bridge (CHB) [3,12,13,14] are the three major multilevel inverter topologies that have received a lot of research interest. Among the three topologies, the CHB multilevel inverter is the most popular due to its simplicity, reliability, modularity, low component count, and high fault tolerance [2,3,7,12,13,14,15]. The modular structure of the H-bridge inverter enables cascading and stacking of inverters for high-power and high-voltage applications. Furthermore, despite failure caused by other cells, the cascaded H-bridge inverter can continue to operate at low power levels [3,12,13]. The disadvantage of the CHB inverter is that it requires a separate DC supply for each module. Thus, to maximise the number of stepped-voltage output levels from a single DC source, researchers have developed a new H-bridge structure by adding a bidirectional switch to the conventional single-phase H-bridge cell [3,12,14,15].

The switching strategies (modulation techniques) used in a power electronic converter are critical to the performance of the converter because they determine the efficiency of the inverter by reducing switching losses and minimising the harmonic content of the output voltage and current. The most difficult aspect of the power-switching strategy is reducing or eliminating the lower-order harmonic using a simple modulation technique [3,4,12,13,14,16]. The harmonic content of the AC-stepped output waveform must be minimised to avoid grid distortion and to achieve maximum energy efficiency [3]. The modulation techniques for multilevel inverters can be divided into two main categories, which include high-frequency and low-frequency (fundamental frequency) switching strategies. The power switches are switched at a low fundamental frequency in the fundamental switching strategy. In contrast, for high-frequency switching, the power switches are switched at a higher frequency range, which consequently pushes the harmonics into a higher frequency range, where they are filtered out by a filter circuit. The two modulation techniques most commonly used by researchers are sinusoidal pulse width modulation (SPWM) [1,2,10,17,18,19,20,21] and selective harmonic elimination (SHE) [3,5,12,13,14,15]. The SHE switching technique falls under the low-switching-frequency category, while the SPWM switching technique falls under the high-switching-frequency category.

In the SHE technique, the switching angles of the power switches in the multilevel inverter circuit are first pre-calculated and pre-defined to eliminate or minimise low harmonic orders (3rd, 5th, 7th, 9th, 11th, 13th, and 17th) that are close to the fundamental frequency. To selectively eliminate specific harmonic orders, a set of transcendental equations must be solved. Newton–Raphson (NR) [13,14,16,22], genetic algorithms (GA) [22], particle swarm optimization (PSO) [3,22,23,24], and evolutionary programming (EP) are examples of algorithms that have been used to solve transcendental equations. With low switching frequency, efficient DC source utilisation, and direct control over low harmonic orders without any filter circuit, the SHE modulation technique has been found to be feasible for a multilevel inverter circuit [12,14,15,24,25]. Therefore, to reduce switching losses, this technique is preferable.

The SPWM technique is widely used in the modulation of conventional inverters and multilevel inverters due to its easy implementation and satisfactory performance [1,2,26,27,28,29]. In contrast to the SHE method, the switching angles for SPWM are determined by comparing the sinusoidal reference signal with carrier waves. SPWM can be divided into different categories according to the generated carrier-wave signals, such as phase-shifted PWM (PS-PWM) and level-shifted PWM (LS-PWM) [1,19,29]. For PS-PWM, the carrier is arranged to cover the whole range of modulation indices, with each carrier phase-shifted by θ. The alternative to PS-PWM is LS-PWM; this technique has three different configurations, known as phase-disposition PWM (PD-PWM), phase-opposition-disposition PWM (POD-PWM), and alternative-phase-opposition-disposition PWM (APOD-PWM) [2,29]. Among these three configurations, the most commonly used LS-PWM is PD-PWM [2]. The main drawback of the SPWM technique is that its average switching frequency and total harmonic distortion (THD) are relatively high, which results in higher switching losses compared to SHE [13,16].

Various design platforms have been used by researchers to implement switching-control algorithms, which include microcontrollers, digital signal processors (DSPs), field-programmable gate arrays (FPGAs), dSPACE, and others [3,17,20]. Previously, many digital switching controllers were implemented using DSPs and microcontrollers. Although microcontrollers and DSPs have gained popularity in power-converter applications because of their easy coding for implementations, they do have some drawbacks, including sampling-rate limitations, lower processing speed, and an inability to handle parallel processing due to their sequential nature of operation [2,3,14,17,20,30]. Recently, FPGA has emerged and is extensively used in power-converter applications due to its parallel-processing features, higher clock-processing speed, and flexibility in hardware integrity [9,10,17,19,20,26,29,31,32,33,34,35,36]. The hardware-parallelism capability of FPGAs significantly improves the processing speed of complex computation algorithms when compared to software-based microcontrollers and DSP solutions [19,20,21,30,34,35]. The greater number of I/O pins on an FPGA device can accommodate the requirement for additional I/O control signals in multilevel-inverter applications [3,37]. As a result, implementing a switching controller in FPGA devices for multilevel-inverter applications provides an excellent solution for efficient hardware design and rapid prototyping [2,3,17].

Several methods have been employed by researchers in the design of SPWM and SHE switching controllers using FPGA devices. Many of the previous and existing design methods for SPWM switching controllers use block RAM (BRAM) or lookup tables (LUT) to store the sampled sine-wave signal and the triangular carrier-wave signal [2,17,18,19,20,21,26,28,38,39]. Recently published work by Sarker et al. (2020 and 2021), for example, used BRAM to store sampled point sine-wave data at a sampling frequency of 4 MHz [2,20]. To reduce the usage of BRAM, only the positive half cycle (0–π) of the sine-wave data were sampled and stored in memory, while the negative half-cycle data were generated by mirroring and inverting the stored positive-cycle data [2,20]. Another recent publication by Juarez-Abad et al. (2021) also used the sampling-points method, where only quarter-wave symmetry of the 60 Hz sine-wave signal was sampled every 4 µs and stored in BRAM, with a size of 1024 × 32 bits [19]. For the triangle carrier-wave signal, Juarez-Abad et al. (2021) also employed the sampling method by using a sampling time of 1 µs and produced 1000 points of sampled data, which were stored in a 1000-word, 32-bit BRAM [19]. However, generating the sine wave or triangular carrier wave by storing each quantized value in memory is not an area-efficient method for hardware implementation and would necessitate more memory usage if the waveforms are implemented in high resolution [20,40]. As a result, a few research papers [27,40,41] proposed the coordination rotation digital computer (CORDIC) algorithm for sine-wave generation in an FPGA-based implementation to reduce the memory requirement.

The CORDIC algorithm provides an iterative method of performing vector rotations by arbitrary angles using very minimal hardware, such as an adder, subtractor, and shifter, thus making it more efficient in terms of resource usage [27]. Ranganathan et al. (2016) proposed a pipelined CORDIC algorithm for sine-wave signal generation to reduce costs and utilisation of hardware resources [27]. Because the design used the pipelined CORDIC method to increase throughput, three sine waves were used in the design at three different phases, which indirectly increased the hardware logic resources and area. Furthermore, the overall design area was not optimised due to the use of a 32-bit RISC softcore processor (Microblaze) as the main controller, an AXI-lite bus for communication with the UART peripheral to receive user input settings on modulation index and switching frequencies, and BRAM for storing calculated sampled points [27]. In other designs by Nikhil et al. (2018), the sine wave was generated using a 32-bit RISC softcore processor (TSK3000A) with floating-point number operations, and the triangular carrier wave was designed using an up-down counter method [42].

For implementation of the SHE digital switching controller, once the optimised switching angles were obtained offline, the switching angles were stored in a LUT for real-time implementation and application [12,13,14]. For example, Halim et al. (2014, 2015, and 2017) used a sinusoidal reference signal and several triggered voltage levels that correspond to the switching angles of the proposed SHE modulation technique [12,13,14]. In this method, the output gating pulses were generated using a sine-wave generator and combinational logic gates. The 50 Hz sine-wave reference signal was sampled at 1 MHz and stored in a LUT [12,13,14]. However, to generate a sine-wave signal, this sampling-point method necessitates 5000 samples, which results in significant memory usage [12,13,14]. In [5], Khalil et al. (2020) employed dual softcore Microblaze processors, four timers, AXI bus, a UART module, BRAM, and GPIO to realise the output gating pulses for the SHE modulation technique after obtaining the switching angles offline from MATLAB Simulink. Although this method was claimed to have a high degree of flexibility in terms of PWM duty-cycle generation, the overall hardware design architecture necessitates the use of significant hardware logic resources.

Based on the previous research findings, most of the existing digital switching controllers for SPWM and SHE techniques employ the sampled-point method for the generation of a reference sinusoidal signal, which requires a large memory size when higher resolution is required. Furthermore, the use of a softcore processor to control and generate the output gating pulses in some designs increases the design complexity and results in significant digital-hardware logic usage. Therefore, there is still room for improvement by reducing hardware complexity and producing an area-efficient design method.

Many DC-AC multilevel inverters have recently been used in solar photovoltaic (PV) system applications to improve power quality and efficiency [6,15,25,28]. It is important to note that in addition to modulation techniques for DC-AC multilevel inverters, there are several concurrent control operations that require continuous monitoring in solar PV system applications, such as maximum power point tracking (MPPT), battery management for charging and discharging processes, and supervisory control to ensure synchronisation of PV output voltage and phase angle with the main grid system [15,25]. The MPPT controller is needed to track and tap maximum power under rapidly changing environmental climates [15,23,25]. A battery-charger controller is required for battery management because it determines when charging activities have reached a state of charge of 100 percent and protects batteries [15,25]. In the PV system, the supervisory controller monitors the PV and grid output voltages on a continual basis to ensure that the PV’s output is in phase with the grid and to maintain a unity power factor [15,25]. Therefore, to have multiple operations running concurrently, high-speed and robust digital controllers are needed. Based on the multiple parallel operations that need to be regulated in the solar PV system, the FPGA device is the most suitable and reliable digital controller to handle this complicated parallel processing precisely and at a higher processing speed than conventional digital controllers, such as DSPs or microprocessors.

Motivated by the advantages provided by the FPGA chip for multilevel inverters in solar PV applications, two types of digital switching controllers, namely SHE and SPWM, were designed using an Intel FPGA board. The primary objective of this research was to design digital switching controllers for a 21-level multilevel inverter with minimal hardware complexity and logic utilisation while maintaining good performance. Thus, rather than relying on complex softcore processors and large amounts of memory (RAM), the SHE switching controller was designed using a simple four-bit finite state machine (FSM) and a 13-bit counter, whereas for the SPWM switching controller, a CORDIC algorithm, a six-bit up-down counter, and a few adders were used to generate reference sine-wave and triangular carrier signals, respectively. In this paper, details of the design methodology and implementation of SHE and SPWM digital switching controllers for 21-level multilevel inverters are explained. The performance of the digital switching controllers was analysed in terms of logic-element utilisation, processing speed, and THD. Initially, the designs of SHE and SPWM switching controllers were hardcoded into digital logic using hardware description language (HDL) Verilog codes. Testbenches were developed to verify the functionality of the designed digital switching controllers using Quartus II Modelsim software (Intel, Santa Clara, CA, USA). For real-time hardware measurement and verification, the designed digital switching controllers were synthesised and downloaded to an Intel FPGA (DE2-115) board (Terasic Inc., Hsinchu, Taiwan). The designed switching controllers were also verified using HDL co-simulation in the MATLAB Simulink (Mathworks, Portola Valley, CA, USA) environment for system-level verification. The 21-level stepped sine-wave output waveform and the THD performance were analysed.

This paper is organised as follows: Section 2 presents the system overview of the 21-level multilevel inverters and the internal architecture of the five-level H-Bridge inverter. The working principles of SHE and SPWM for the 21-level multilevel inverter are illustrated and explained briefly. Section 3 elucidates the design methodology and implementation of SHE and SPWM digital switching controllers. Each design method is explained in detail, starting from designing the switching controller in the HDL to system-level verification using HDL co-simulation in MATLAB Simulink. Section 4 discusses synthesised logic gates, register-transfer-level (RTL) simulation results obtained from HDL functional simulation, hardware measurement results, and system-level verification using HDL co-simulation in MATLAB Simulink. In this section, the performance of the designed SHE and SPWM digital switching controllers is analysed, discussed, and compared with other research work. Finally, Section 5 presents the conclusion of the research work.

## 2. System Overview of 21-Level Cascaded H-Bridge Multilevel Inverter

**Figure 1:**
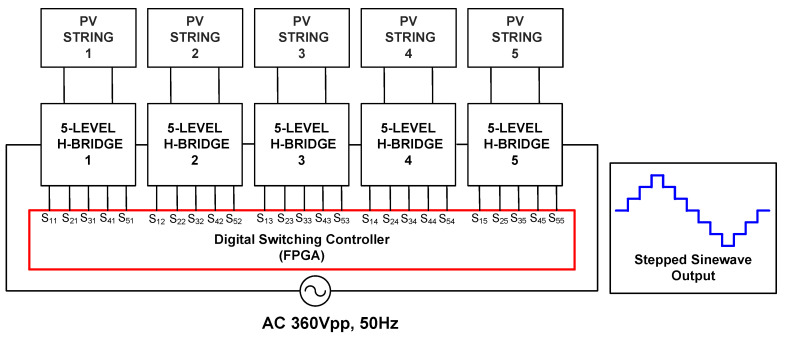
System overview of the 21-level cascaded H-bridge multilevel inverter.

**Figure 2:**
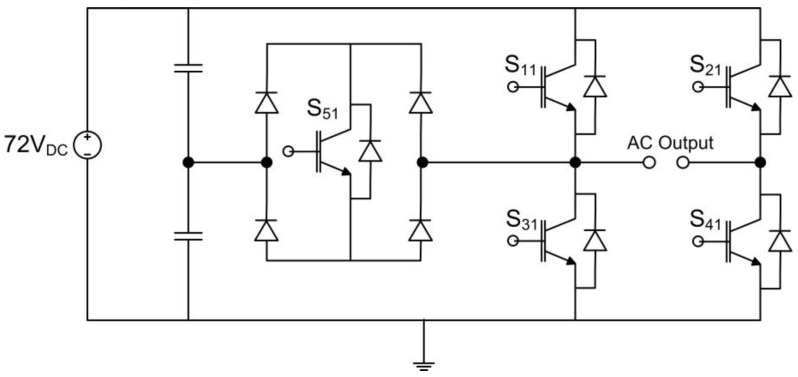
Example of five-level H-Bridge 1 circuit using photovoltaic (PV) string as the DC supply voltage (72 V_DC_).

The overall system overview of a 21-level multilevel inverter, as depicted in Figure 1, mainly consists of a digital switching controller and five cascades of five-level H-bridge circuits (H-Bridge 1 to H-Bridge 5). The digital switching controller was designed and implemented using an Intel FPGA (DE2-115) board. The five-level H-bridge circuit consists of five solid-state insulated-gate bipolar transistor (IGBT) switches, as illustrated in Figure 2. The supply voltage of this H-bridge circuit was 72 V_DC_, which was from the PV string. To turn ON the IGBT switches, the output signals from the digital switching controller (S_11_–S_55_) were first amplified to 12 V by optocoupler gate drivers (HCPL-3020). The basic operation of the 21-level multilevel inverter depends on the switching pattern of the IGBT switches, generating a full cycle of AC-stepped output waveforms.

Table 1 lists the switching patterns required to produce the stepped sine-wave output waveform of 50 Hz for the 21-level cascaded H-bridge multilevel inverter using the SHE digital switching controller. Since each five-level H-bridge inverter has a supply voltage of 72 V_DC_, the output voltage in the table needs to be multiplied by 72 V. Therefore, the final stepped sine-wave output voltage for five cascaded H-bridges ranges from 360 V_P_ to −360 V_P._

**Figure 3:**
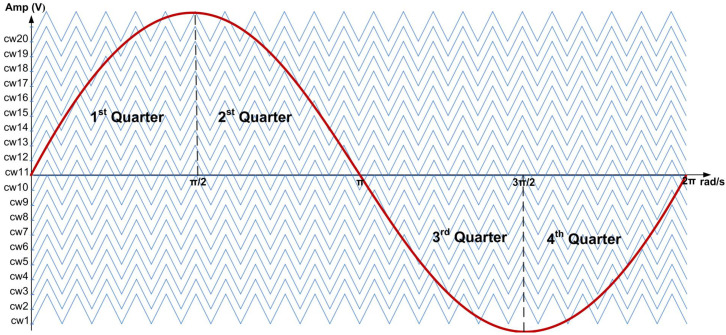
Symmetric disposition of multicarrier phase-disposition PWM (PD-PWM).

**Figure 4:**
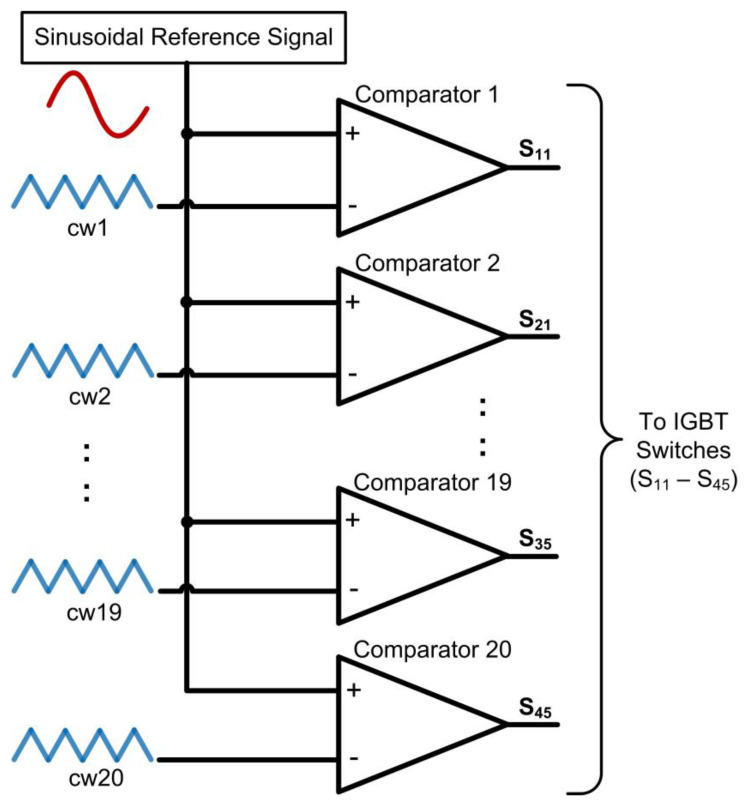
Switching generation using PD-SPWM technique for one-phase, 21-level multilevel inverter.

For the SPWM technique, the required number of carriers for the PD-PWM method to generate an N-level inverter is N-1 [18]. Therefore, for the 21-level SPWM inverter, 20 levels of carrier waves are needed. Figure 3 illustrates 20 carrier waves (cw1-cw20) using the symmetric distance of the PD-PWM method. These carrier waves (cw1-cw20) are continuously compared with the reference sinusoidal wave using comparator circuits, as illustrated in Figure 4. Therefore, 20 output gating pulses (S_11_ to S_45_) are generated to turn ON the IGBT switches and produce the 21-level stepped sine-wave output waveform.

The output gating pulses of five IGBT switches in each H-bridge circuit for SHE and SPWM switching controllers are listed in Table 2. Note that the “X” notation represents the number of H-bridge circuits. For the SHE switching controller, five output gating pulses (S_1X_ to S_5X_) were connected to five IGBT switches in each H-bridge circuit. However, for the SPWM switching controller, only four output gating pulses (S_1X_ to S_4X_) were connected to four IGBT switches in each five-level H-bridge circuit. The fifth IGBT switch was not used or activated in any H-bridge circuits for the SPWM switching controller.

The IGBT switches in each H-bridge circuit are turned ON and OFF in pairs to allow current to flow in both directions (positive and negative cycles). For example, referring to Figure 2, during a positive cycle, switches S_11_ and S_41_ are turned ON, while switches S_21_ and S_31_ are turned OFF. During a negative cycle, switches S_21_ and S_31_ are turned ON, while switches S_11_ and S_41_ are turned OFF. For the SHE switching controller, switch S_51_ is always turned ON for a certain duration for both cycles. The dead-time delay between the upper (S_1X_ or S_2X_) and lower (S_3X_ or S_4X_) IGBT switches for each side of the H-bridge circuit was considered at the initial design stage of the SHE and SPWM switching controllers. The dead-time delay prevents upper and lower IGBT switches on the same side of the H-bridge circuit from turning ON at the same time, and it also protects IGBT switches from damage caused by a short circuit across the DC link [20,43]. For example, before the upper IGBT switch on each side of the H-bridge circuit (S_1X_ or S_2X_) is turned ON, it must wait for the lower IGBT switch (S_3X_ or S_4X_) to first be turned OFF after a certain time delay, which is known as the “dead time”. In contrast, when the lower IGBT switch (S_3X_ or S_4X_) is turned ON, it must wait for the upper IGBT switch (S_1X_ or S_2X_) to first be turned OFF after a certain dead-time delay. In the previous hardware-measurement setup, the output gating pulses from the digital switching controller were connected to the IGBT (IKP10N60T) switches via optocouplers (HCPL-3020), which act as drivers to turn ON the IGBT (IKP10N60T) switches by amplifying the output gating pulses to a higher voltage (12 V) [3,15,25]. The total delay time for each IGBT (IKP10N60T) switch to turn ON and OFF and the propagation-delay difference of the optocoupler (HCPL-3020) driver were taken into consideration for the calculation of the dead-time duration. Therefore, dead time was determined as shown in Equation (1) [20,43]:

(1) $$T_{\mathrm{dead}}=\left[\left(T_{d\_\mathrm{OFF}\_\max\;}-T_{d\_\mathrm{ON}\_\min\;}\right)\;+\left(T_{\mathrm{pdd}\_\max\;}-T_{\mathrm{pdd}\_\min\;}\right)\right]\times 2.5$$

where (T_d_OFF_max_ − T_d_ON_min_) is the difference between the IGBT (IKP10N60T) switch’s maximum turn-off delay time (233 ns) and its minimum turn-on delay time (10 ns) [44]. The minimum (T_PDD_MIN_) and maximum (T_PDD_MAX_) propagation-delay differences of the optocoupler (HCPL-3020) are −0.5 µs and 0.5 µs, respectively. A constant value of 2.5 was used as a safety factor [20,43]. By referring to the datasheets, the calculated minimum dead time for both the IGBT (IKP10N60T) [44] and optocoupler (HCPL-3020) [45] components was 3.05 µs.

Details of design implementation for SHE and SPWM digital switching controllers will be further elucidated in the following sections.

## 3. Materials and Methods

This section discusses the design methodology and implementation of the SHE and SPWM digital switching controllers using the Intel FPGA (DE2-115) board. In this research work, there are three main design phases that need to be executed for implementation of the FPGA-based SHE and SPWM digital switching controllers. Firstly, the process started with designing the SHE and SPWM digital switching controllers using hardware description language (HDL) Verilog codes for implementation of digital hardware logic. Testbenches were also developed in HDL Verilog codes to verify the design functionality. Secondly, the designed controllers were synthesised and downloaded onto the Intel FPGA (DE2-115) board for real-time hardware measurement and verification. Finally, for system-level verification, the designed SHE and SPWM digital switching controllers were imported into MATLAB Simulink and connected to five cascaded H-bridge models to generate the 21-level stepped sine-wave output waveform using the HDL co-simulation method. The performance of the designed digital SHE and SPWM switching controllers was analysed in terms of their hardware logic requirements, maximum processing speed (F_max_), execution speed, and total harmonic distortion (THD). The following sub-sections provide detailed explanations of each design phase.

### 3.1. SHE Digital Switching Controller Design Method

**Figure 5:**
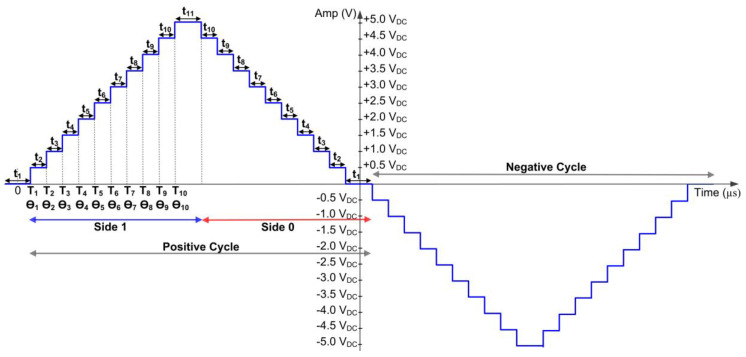
Location of switching time (T_1_–T_10_), switching angles (θ_1_–θ_10_), and step-time duration (t_1_–t_11_) in the stepped sine-wave output waveform.

For the SHE digital switching controller, the design process started with finding the optimised switching angles (θ_1_–θ_10_) for the 21-level multilevel inverter, as depicted in Figure 5. The locations of the switching times (T_1_–T_10_) for the corresponding switching angles (θ_1_–θ_10_) with respect to the full cycle of the stepped sine-wave output waveform are also illustrated in Figure 5. The switching angles were pre-calculated offline using the modified hybrid PSO (MhyPSO) algorithm in MATLAB software. The MhyPSO algorithm was designed to produce optimised switching angles (θ_1_–θ_10_) with reduced odd harmonic content [15,46]. Additionally, the dead times between the upper and lower IGBT switches were also considered at the initial stage of MhyPSO algorithm development. To avoid short circuits, the MhyPSO algorithm ensures that the upper (S_1X_ or S_2X_) and lower (S_3X_ or S_4X_) IGBT switches are not turned ON at the same time during the dead time. Detailed information on the MhyPSO algorithm for the SHE modulation technique can be found in [15,46].

The obtained optimised angles (θ_1_–θ_10_) were first converted into switching times (T_1_–T_10_), as shown in Equation (2).

(2) $$T\;=\left(\frac{\theta }{180}\right)\times 10\;\mathrm{ms}$$

Table 3 lists the conversion of the optimised switching angles (θ_1_–θ_10_) obtained from the previous research work [15,46] into switching times (T_1_–T_10_).

Thereafter, the step-time durations (t_1_–t_11_) were calculated offline using the switching times (T_1_–T_10_), as shown in Table 4 and illustrated in Figure 5. The pre-calculated step-time durations (t_1_–t_11_) were used as references by the SHE digital switching controller to produce the output gating pulses according to the switching patterns, as listed in Table 1.

It is important to note that the 21-level stepped sine-wave output signal is a symmetrical sine wave that consists of positive and negative cycles. Therefore, the step-time durations (t_1_–t_11_) are similar for both cycles (positive and negative). The only difference is the direction of voltage or current (positive or negative). As depicted in Figure 5, each cycle of the sine wave has a quarter-wave symmetry shape with a step-time duration (t_1_–t_11_). The advantage of this quarter-wave symmetry shape of the sine wave in each cycle is that it minimises the hardware logic resources by using similar step-time durations (t_1_–t_11_) for the mirrored shape. Therefore, in this design, each cycle of the step sine wave is divided into two sides: Side 1 and Side 0. Side 1 covers step-time durations (t_2_–t_11_), while Side 0 covers step time (t_10_–t_1_). Detailed calculations for all step-time durations (t_1_–t_11_) with their corresponding stepped output levels are listed in Table 4. The digital implementation of this quarter-wave-symmetry sine wave is explained in the following sub-section.

#### SHE Design Architecture and Protocol

**Figure 6:**
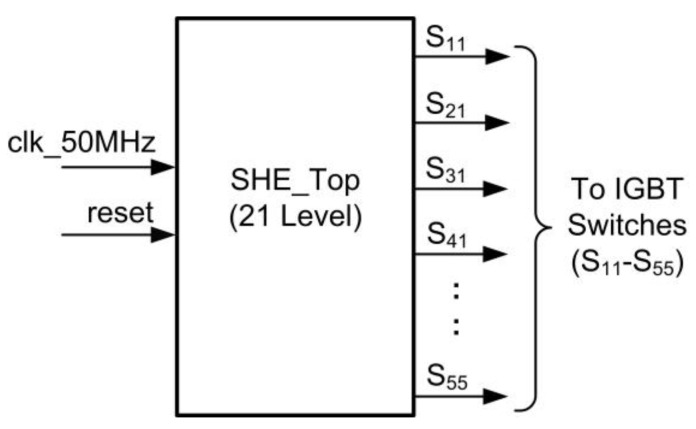
Top level of the SHE digital switching controller.

**Figure 7:**
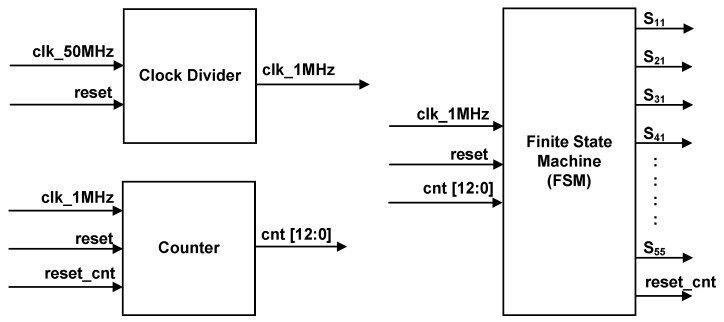
Internal architecture of the SHE digital switching controller.

Figure 6 depicts the top level of the SHE digital switching controller. The internal architecture of the SHE digital switching controller is depicted in Figure 7.

The design architecture of the SHE digital switching controller mainly consists of three sub-modules, which include a clock divider, a 13-bit counter, and a 4-bit finite state machine (FSM). The clock-divider sub-module divides and down-converts the 50 MHz system clock (“clk_50M”) from the FPGA (DE2-115) board into a clock with a low frequency of 1 MHz (“clk_1MHz”). The “clk_1MHz” clock is used by the counter sub-module for the count-up process with a time resolution of 1 µs. After the system is powered ON and reset, the counter sub-module starts to count-up and provides a 13-bit counted output value (“cnt [12:0]”) to the FSM sub-module. The FSM sub-module controls the generation of a stepped sine-wave output waveform by monitoring the step-time duration (t_1_–t_11_) for the positive and negative cycles. The 13-bit counted value (“cnt [12:0]”) is used by the FSM sub-module to compare with the referenced step-time durations (t_1_–t_11_), as listed in Table 4. Once the counted value (“cnt [12:0]”) reaches the referenced step-time duration (t_1_–t_11_), the FSM activates an internal reset signal (“reset_cnt”) to reset the counter’s internal registers to a value of “0”. The FSM sub-module provides 25 output gating signals (S_11_–S_55_), which are connected to the 25 IGBT switches in the five cascaded H-bridge circuits, as depicted in Figure 1.

**Figure 8:**
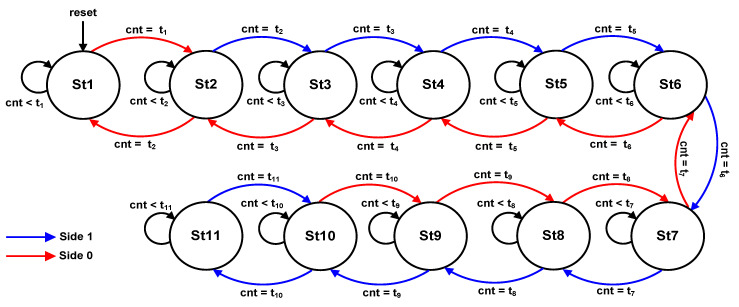
State diagram of the SHE digital switching controller.

As illustrated in Figure 5, the stepped sine wave for each cycle consists of Side 1 for step-time duration t_2_–t_11_ and Side 0 for step-time duration t_10_–t_1_. The stepped sine-waveform protocol of the FSM sub-module is depicted in Figure 8.

In this design, only eleven states (St1–St11) are required to generate a complete cycle of the 21-level stepped sine-wave output waveform. Therefore, only four bits are required to generate the eleven states. As illustrated in Figure 8, the FSM uses the 13-bit counted value (“cnt [12:0]”) to compare with the referenced step-time durations (t_1_–t_11_) to control the movement of the next states. Additionally, the FSM also uses an internal control signal (“side”) to represent either Side 1 (logic HIGH) or Side 0 (logic LOW). This internal “side” signal is used by the FSM to determine the direction of the state movement. When the internal “side” signal is logic HIGH, the FSM executes states St2 to St11, as indicated by the blue arc arrow (Side 1). When the internal “side” signal is logic LOW, the FSM executes states St10 to St1, as indicated by the red arc arrow (Side 0). In each state, the FSM produces 25-bit output gating signals (S_11_–S_55_), which are connected to 25 IGBT switches in the five cascaded H-bridge circuits.

After the system is powered ON and reset, the FSM enters state St1 and remains in this state for a step-time duration of t_1_ (240 µs). When the counted value (“cnt [12:0]”) is equal to t_1_, the FSM moves to the next state, St2, and activates a “reset_cnt” signal. The “reset_cnt” signal resets the internal counter register (“cnt [12:0]”) in the counter sub-module and repeats the count-up process again. The FSM remains in state St2 until the 13-bit counted value (“cnt [12:0]”) reaches the step-time duration of t_2_ (339 µs). Then, the FSM moves to state St3, and so forth. The process of comparing the 13-bit counted value (“cnt [12:0]”) with each dedicated step-time duration (t_1_–t_11_) at each state is repeated until state St11, as depicted in Figure 8. Thus, a complete switching pattern (S_11_–S_55_) for Side 1 (blue arc arrow) of the positive cycle is generated, as illustrated in Figure 8. From state St11, the FSM moves in a backward direction to state St10 until St1, as indicated by the red arc arrow, to execute Side 0 for step-time durations of t_10_–t_1_. When the FSM finally reaches state St1, an internal polarity signal is toggled for the negative-cycle execution. Then, the process of step-time duration for Side1 and Side0 is repeated but with the opposite polarity (negative cycle). This polarity signal is used as a reference signal by each state to produce the respective switching patterns or logic values (S_11_–S_55_) accordingly. The repetition of the loop-cycle for similar consecutive state movements (St1–St11) in the FSM sub-module results in the continuous generation of positive and negative switching patterns at the output gating signals (S_11_–S_55_). These output gating pulses (S_11_–S_55_) activate the 25 IGBT switches in the five cascaded H-bridge circuits, resulting in a continuous 21-level stepped sine-wave output waveform.

Table 5 tabulates the details of states with their respective polarity cycles and switching output gating patterns (S_11_–S_55_) in the FSM sub-module. Different output-switching patterns are produced for positive (pos) and negative (neg) cycles.

During the design phase of the SHE digital switching controller, functional simulations were conducted to ensure that the output gating pulses produced accurate step-time durations, which had been pre-calculated according to the optimised switching angles.

### 3.2. SPWM Digital Switching Controller Design Method

**Figure 9:**
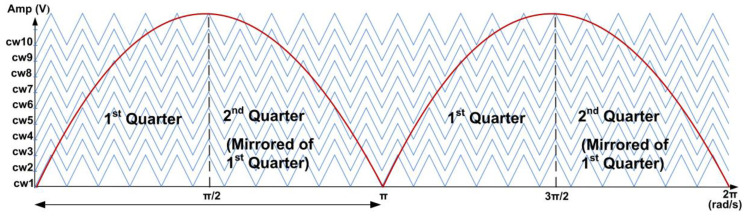
Ten symmetric dispositions of multicarrier phase-disposition PWM (PD-PWM) for a sinusoidal reference signal with a positive cycle only.

In the design of the SPWM digital switching controller, the CORDIC algorithm was used to generate the reference sine-wave signal because it significantly reduces the memory storage requirement compared to the conventional sampled-points method [27,40,41]. In this design, the rotation mode of the CORDIC algorithm was implemented to calculate the sine value of the phase angle between 0° and 90° instead of 0° to 360°. To generate a full-cycle sinusoidal wave, mirror properties of the sine wave (mirroring and inverting the 0° to 90° values for the 2nd, 3rd, and 4th quarters, as shown in Figure 3) were used, as illustrated in Figure 9. By exploiting the symmetry property of the sinusoidal wave, a complete sinusoidal waveform can be generated. Initially, the phase angle is incremented from 0° (0 rad) until it reaches 90° (π/2 rad) for the first quarter. Then, the phase angle is decremented to 0° (0 rad) for the second quarter (mirror of the first quarter). A similar process is repeated for the negative cycle.

In the level-shifted PD-PWM technique, (N − 1)/2 carrier signals are needed for the half-cycle of the N-level inverter [35]. Therefore, in this design, only ten triangular carrier-wave signals were required, instead of 20, since only the positive cycle of the reference sine wave was used for comparison. The maximum amplitude for the reference sine-wave and carrier-wave signals was 255. The amplitude differences among the ten carrier-wave signals were 25, except for the last carrier (cw10), which had an amplitude difference of 24. The first carrier-wave signal (cw1) was designed using a 6-bit up-down counter, for which the maximum value was 31. The other carrier-wave signals were generated using an adder function, which added the first amplitude carrier (cw1) with an offset value, as shown in Table 6. The ranges of triangular amplitude values (start–max–end) are also shown in Table 6.

#### SPWM Design Architecture and Protocol

**Figure 10:**
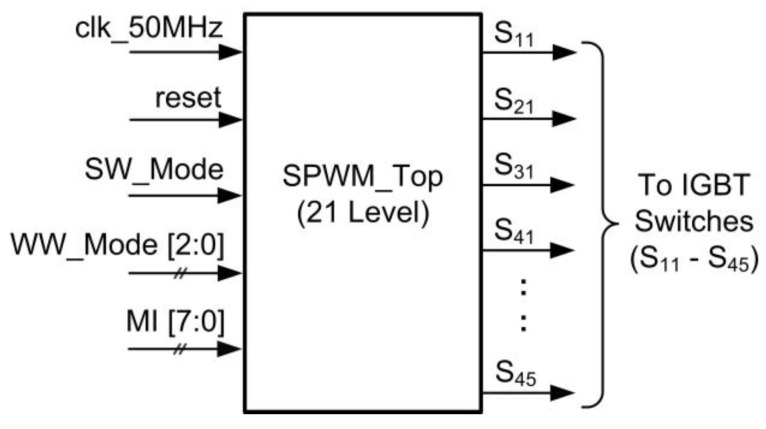
Top level of the SPWM digital switching controller.

**Figure 11:**
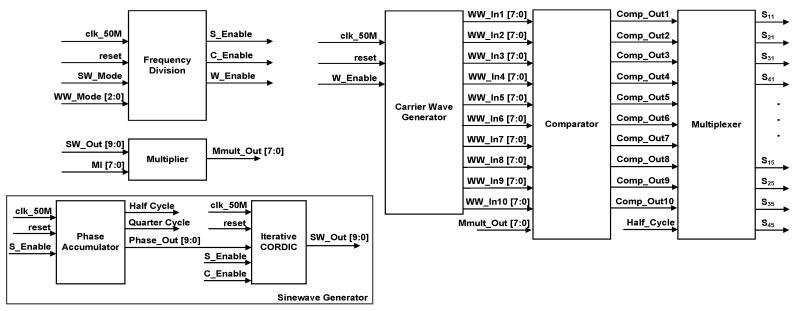
The internal architecture of the SPWM digital switching controller.

Figure 10 depicts the top level of the SPWM digital switching controller. The internal architecture of the SPWM digital switching controller is depicted in Figure 11. The design architecture of the SPWM digital switching controller mainly consists of a frequency division, a sine-wave generator, a multiplier, a carrier-wave generator, a comparator, and a multiplexer.

The FPGA (DE2-115) board’s 50 MHz system clock (“clk_50M”) was used as a master clock for the frequency-division, sine-wave-generator, and carrier-wave-generator sub-modules. The frequency-division sub-module generates three enabling signals for the carrier-wave-generator and sine-wave-generator sub-modules, which include “W_Enable”, “S_Enable”, and “C_Enable” at different rates of frequencies. The carrier-wave generator generates ten carrier-wave signals (“WW_In1 [7:0]” to “WW_In10 [7:0]”) with a few ranges of frequencies (1 kHz to 800 kHz) based on external user-input settings (“WW_Mode”). The “W-Enable” signal is used to control the internal up-down counter operation of the carrier-wave-generator sub-module.

The sine-wave-generator sub-module generates sinusoidal reference frequencies of either 50 or 60 Hz based on an external user-input setting (“SW_Mode”). The sine-wave generator consists of phase-accumulator and iterative CORDIC sub-modules. The “S_Enable” and “C_Enable” signals control the activation of internal counters, shifters, and registers in the sine-wave-generator sub-module. The phase accumulator generates new phase values (“Phase_out [9:0]”) ranging from 0 to 1.5703125 radians (0° to 90°), which are connected to the iterative CORDIC sub-module. In this work, the resolution of phase value is 0.0061581 radians (1.5703125 radian is divided by 255) for an 8-bit sinusoidal reference signal. The phase accumulator also generates a “Quarter_Cycle” signal, which toggles its logic value every 90° (π/2 rad). The “Quarter_Cycle” is used as a reference to increase or decrease the phase value (“Phase_out [9:0]”). The phase value (“Phase_out [9:0]”) is incremented or decremented by the resolution of 0.0061581 radians when the “Quarter_Cycle” signal is at logic LOW (1st quarter) or at logic HIGH (2nd quarter), respectively. The iterative CORDIC sub-module uses the generated phase value (“Phase_out [9:0]”) as an initial phase input to generate the final sine-wave value (“SW_Out [9:0]”) after eight iterations using the CORDIC algorithm. Although the CORDIC algorithm is not a straightforward method to generate the sinusoidal reference signal, it enables the usage of a small LUT by storing only eight arctan angles (θ = 45.0°, 26.6°, 14.0°, 7.1°, 3.6°, 1.8°, 0.9°, and 0.4°), compared to the sampled points method. By exploiting the capability of bi-directional angles of rotation (clockwise or anticlockwise) in the CORDIC algorithm, the angles can be varied from +99.4° to −99.4°. Details of the implementation of the CORDIC algorithm to generate the sinusoidal reference signal can be found in [40,41].

The generated sinusoidal reference signal (“SW_Out [9:0]”) is then multiplied with the modulation index (“MI [7:0]”) ranging from 0.1 to 1 in the multiplier sub-module. The multiplied sine-wave output signal (“Mmult_out [7:0]”) is used as a reference signal for the comparator sub-module. The reference sinusoidal signal (“Mmult_out [7:0]”) is then compared with ten carrier-wave signals (“WW_In1 [7:0]” to “WW_In10 [7:0]”) to produce ten comparator output gating signals (“Comp_Out1–Comp_Out10”). For a full cycle of stepped sine-wave output waveforms, a “Half_Cycle” signal is used as a reference by the multiplexor sub-module to generate 20 SPWM output gating pulses (“S_11_” to “S_45_”). The “Half_Cycle” signal, which is generated by the phase-accumulator sub-module, toggles its logic value every 180° (π rad). When the “Half_Cycle” signal is at logic HIGH, the five positive pairs of SPWM output gating pulses (“S_11_”, “S_41_”, “S_12_”, “S_42_”, “S_13_”, “S_43_”, “S_14_”, “S_44_”, “S_15_”, and “S_45_”) are generated for a positive cycle. When the “Half_Cycle” signal is at logic LOW, the five negative pairs of SPWM output gating pulses (“S_21_”, “S_31_”, “S_22_”, “S_32_”, “S_23_”, “S_33_”, “S_24_”, “S_34_”, “S_25_” and “S_35_”) are generated for the negative cycle. These SPWM output gating pulses are connected to the 20 IGBT switches to generate the final 21-level stepped sine-wave output waveform. For the realisation of dead-time delay between the upper (“S_1X_” or “S_2X_”) and lower (“S_3X_” or “S_4X_”) IGBT switches on each side of the H-bridge circuit, the “Half_Cycle” signal ensures that only positive pairs of IGBT switches are turned ON during the positive cycle, while negative pairs of IGBT switches are turned OFF, and vice versa for the negative cycle. Once the SPWM design was completed, functional simulations were conducted to verify that the output-switching signals could produce positive and negative gating pulses accordingly for the 50 Hz stepped sine-wave generation.

### 3.3. Hardware-Measurement Setup

**Figure 12:**
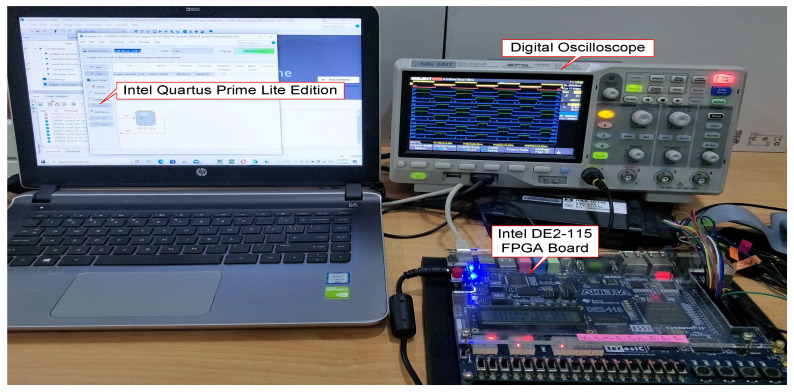
Hardware-measurement setup using an Intel field-programmable gate array (FPGA) (DE2-115) board and a digital oscilloscope.

After the functional behaviours of the designed SHE and SPWM switching controllers were verified to comply with the specified output gating pulse patterns and timing durations, the designs were synthesised and downloaded onto the Intel FPGA (DE2-115) board for real-time hardware verification. Figure 12 depicts the hardware-measurement setup of the designed SHE and SPWM digital switching controllers for real-time verification purposes. The hardware setup mainly consists of an Intel FPGA (DE2-115) board and a digital oscilloscope (SIGLENT Technologies, Shenzhen, China). For measurement purposes, the output gating pulses of the SHE digital switching controller (S_11_ to S_55_) and the SPWM digital switching controller (S_11_ to S_45_) were connected from the FPGA board to the digital oscilloscope. It is to be noted that for real-time hardware verification, only the output gating pulses of the SHE and SPWM digital switching controllers were measured and verified with the functional simulation results.

### 3.4. System-Level HDL Co-Simulation Development for Digital Switching Controllers

**Figure 13:**
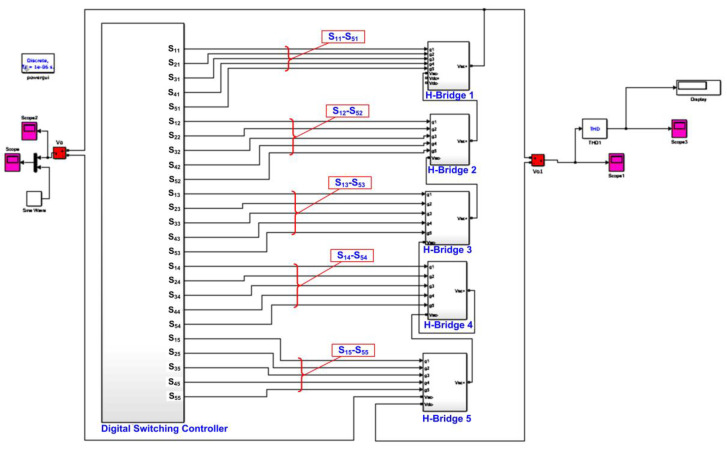
Example of system-level hardware description language (HDL) co-simulation development for the SHE digital switching controller using five cascaded H-bridge multilevel inverter model in MATLAB Simulink.

Figure 13 depicts the system-level settings of the 21-level cascaded H-bridge multilevel inverter model in the MATLAB Simulink environment. For system-level verification and performance analysis, the HDL codes were first imported into MATLAB Simulink. This HDL system-level verification in MATLAB Simulink is also known as HDL co-simulation. The output gating pulses from the designed SHE and SPWM digital switching controllers were connected to the IGBT switches to generate the final stepped sine-wave output waveform. For analysis purposes, the stepped sine-wave output waveform and the THD percentage were observed.

## 4. Results and Discussions

This section discusses the results of synthesised SHE and SPWM digital switching controllers using the Intel FPGA (Cyclone IV E) chip, register transfer-level (RTL) simulation results in a digital-simulator timing waveform, real-time hardware-measurement results, and system-level verification using HDL co-simulations in MATLAB Simulink. The results are explained sequentially in separate sub-sections.

### 4.1. Synthesized Logic Gates of Digital Switching Controllers and Comparative Analyses

**Figure 14:**
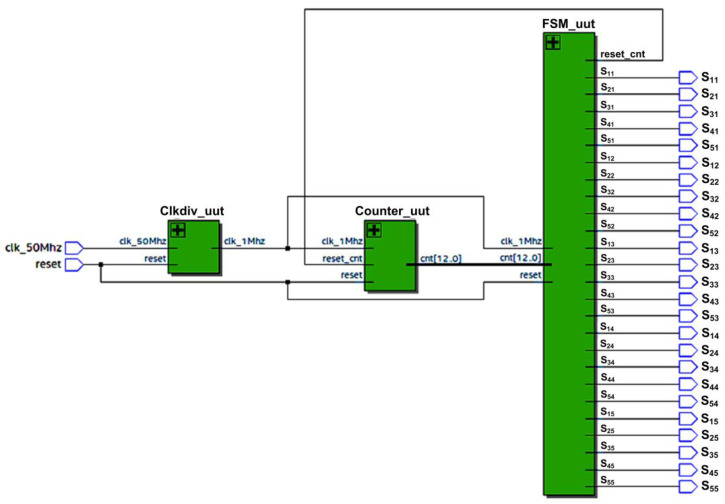
Schematics of the synthesised SHE digital switching controller using the Intel FPGA (Cyclone IV E) chip.

**Figure 15:**
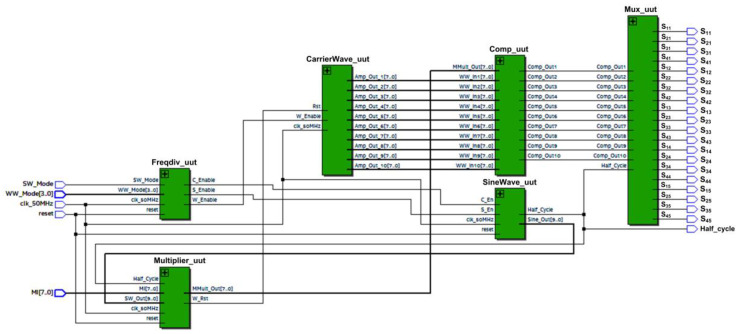
Schematics of the synthesised SPWM digital switching controller using the Intel FPGA (Cyclone IV E) chip.

Figure 14 depicts the schematics of the synthesised SHE digital switching controller using Quartus II software for the Intel FPGA (Cyclone IV E) chip. The internal architecture of the synthesised SHE digital switching controller mainly consists of the clock-division (Clkdiv_uut), counter (Counter_uut), and FSM (FSM_uut) sub-modules, as shown in Figure 14. Figure 15 depicts the schematics of the synthesised SPWM digital switching controller using Quartus II software for the Intel FPGA (Cyclone IV E) chip. The internal architecture of the synthesised SPWM digital switching controller mainly consists of the frequency-division (Freqdiv_uut), carrier-wave-generator (CarrierWave_uut), sine-wave-generator (SineWave_uut), multiplier (Multplier_uut), comparator (Comp_uut), and multiplexer (Mux_uut) sub-modules, as shown in Figure 15.

Details on the FPGA family name, device types, utilisation of logic elements, and the maximum frequency (F_max_) at which the design could operate are listed in Table 7.

According to the synthesised results shown in Table 7, the total number of logic elements used for the designed SHE digital switching controller was 186 (<1% utilisation), the total number of registers was 59, the total number of pins was 27 (5% utilisation), the total number of memory bits was 0 (0% utilisation), and the total number of embedded multipliers was 0 (0% utilisation). The maximum frequency (F_max_) at which the system could operate at a high temperature (85 °C) was 198.06 MHz. For the SPWM digital switching controller, the total number of logic elements in the synthesised design was 369 (<1% utilisation), the total number of registers was 108, the total number of pins was 36 (7% utilisation), the total number of memory bits was 0 (0% utilisation), and the total number of embedded multipliers was one (<1% utilisation). The maximum frequency (F_max_) at which the system could operate at a high temperature (85 °C) was 152.25 MHz.

The SPWM digital switching controller’s logic elements and registers are almost double those of the SHE digital switching controller. Additionally, the SPWM digital switching controller utilises a total of 2% more pins and one embedded multiplier. The maximum frequency of the SHE digital switching controller is 6% higher than that of the SPWM when the 50 MHz clock is used as a reference. Due to the design complexity of the SPWM digital switching controller compared to the SHE controller, it is expected that the SPWM controller requires more hardware logic resources, which consequently results in a slightly lower maximum frequency (F_max_).

The execution speed of the SHE digital switching controller was further compared with that observed in previous research work that used a microcontroller (PIC16F877A) as a design platform [15,25]. Table 8 lists the details of the implementation of the SHE digital switching controller in the FPGA (Cyclone IV E) chip and microcontroller (PIC16F877A).

For a complete cycle of stepped sine-wave output waveforms, the proposed SHE digital switching controller requires 40 states, in which the loop-cycle of 11 states (St1 to St11), as depicted in Figure 8, is executed twice to obtain one complete stepped sine-wave output waveform (equivalent to 40 step-time duration as shown in Figure 5). Therefore, the total execution time is 40 µs when referring to the FSM clock frequency of 1 MHz. For the SHE digital switching controller implemented in the microcontroller (PIC16F877A), 868 machine cycles were required to generate one complete cycle of stepped sine waves [25]. Since the clock frequency used for the PIC16F877A was at 4 MHz, the calculated period of a machine cycle was 1 µs [3]. Thus, the required execution time is 868 µs (868 × 1 µs). From the tabulated results, it is observed that the execution speed of the SHE digital switching controller implemented in the FPGA (Cyclone IV E) chip is 95.39% faster compared to that implemented in the microcontroller (PIC16F877A) when FSM clock frequency (1 MHz) is used. However, when the maximum clock frequency is used (F_MAX_ = 137.99 MHz), the execution speed of the SHE digital switching controller implemented in the FPGA increases to 99.97% when compared to that implemented in the microcontroller (PIC16F877A). These execution-speed analyses proved that the digital switching controller implemented in the FPGA (Cyclone IV E) chip is much faster than that implemented in the conventional microcontroller (PIC16F877A).

As mentioned earlier, many of the existing SHE and SPWM switching controllers require the use of large BRAM to store the sampled-points data and complex softcore processors [2,12,13,17,18,19,20,21,26,27,28,38,39,42]. In contrast, our proposed SPWM design uses only a small LUT to store eight arctan angles for sine-wave generation, one six-bit up-down counter, and a few adders for carrier-wave generation. Meanwhile, for the SHE controller, our proposed method requires only a four-bit FSM and a 13-bit counter.

Table 9 lists a few examples of hardware-logic design methods and synthesised logic gates from other research work as well as from our work. As can be seen from the table, most of the existing modulation techniques (SPWM or SHE) proposed by other researchers require the use of large memory elements (ROM or RAM) to store the sine-wave sampled-points data [2,12,13,14,17,19,20,27]. In some designs, such as those by Nikhil et al. (2018) [42], Ranganathan et al. (2016) [27], and Khalil et al. (2020) [5], softcore processors embedded in the FPGA device were used as the main controller for the switching operations. It is important to note that although the design was implemented using an FPGA device, a softcore processor (32-bit RISC Microblaze or 32-bit RISC TSK-3000A) was used to execute the switch-controlling processes [5,27,42]. The use of softcore processors not only requires significant hardware logic resources but also makes the system slower due to the sequence-controlling operation (similar to DSPs or microcontrollers) compared to a customised digital design using the FSM. Although the customised FSM design also works in sequence, it is much faster compared to the softcores or conventional controllers (DSPs or microcontrollers) because the FSM can change its state immediately if there is a change in the input, and it does not need to fetch and decode instructions to change states.

Due to different internal LUT architectures for different FPGA technologies, an exact one-to-one comparative analysis is not possible. Thus, only an approximate comparative analysis between the Xilinx and Intel FPGA technologies can be performed by referring to logic cells (LCs) and logic elements (LEs) from each technology, respectively. For analysis purposes, the LCs for Xilinx FPGA technology and LEs for Intel FPGA technology are listed in Table 9. The slices data are converted into LCs for Xilinx FPGA technology by referring to the Xilinx Spartan 6 and Spartan 3 datasheets [47,48,49]. From the synthesised logic gates listed in Table 9, it is observed that the number of LCs or LEs reported in other research work is quite high, and the percentage of utilisation for LCs or LEs is greater than 1%. For the SPWM modulation technique, our designed SPWM digital switching controller has the smallest number of LEs (369), with a utilisation percentage of less than 1% when compared to those reported in other research work. Similarly, for the SHE modulation technique, our designed SHE digital switching controller has the smallest number of LEs (186), with a utilisation percentage of less than 1% when compared to those reported in other research work.

In conclusion, when compared to other research work, the proposed SHE and SPWM digital switching controllers are significantly simpler in terms of design architecture and do not require a large amount of hardware logic resources, which account for less than 1% of the total logic elements. Furthermore, our proposed SHE and SPWM digital switching controllers produce the greatest number of levels (21-level) for the multilevel inverter, despite their simplified design architectures and having the fewest hardware logic resources.

### 4.2. RTL Simulation Results of SHE and SPWM Digital Switching Controllers

The RTL simulation results were conducted using Quartus II Modelsim software to verify the designed SHE and SPWM digital switching controllers’ functionality. Details of each switching controller’s functionality are explained in the following sub-sections.

#### 4.2.1. RTL Simulation Results of SHE Digital Switching Controller

**Figure 16:**
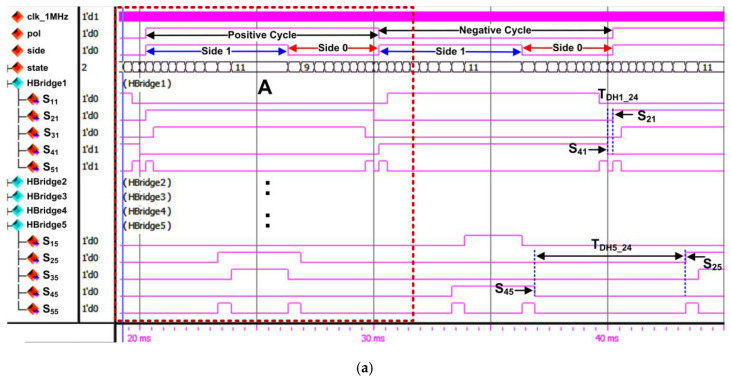

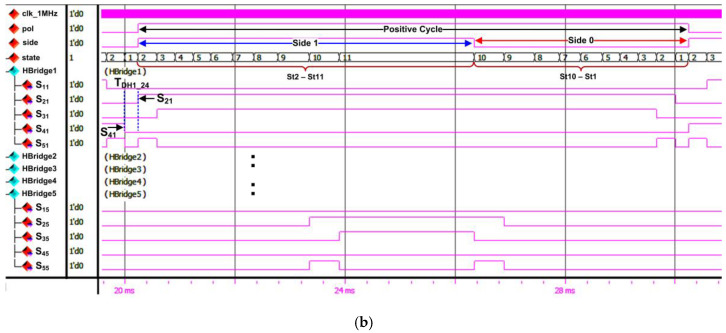
Register transfer-level (RTL) simulation results of the SHE digital switching controller: (**a**) Overall operation of the SHE digital switching controller; (**b**) Zoomed view of marked area A.

Figure 16a depicts the overall RTL simulation results for the SHE digital switching controller. As shown in the simulation result, there is an internal polarity (“pol”) signal to indicate the positive or negative cycle of the stepped sine-wave output waveform and a “side” signal to indicate Side 1 or Side 0 of the waveform. The duration for each polarity cycle (positive or negative) is 10 ms. Therefore, the total duration to generate the overall stepped sine-wave output waveform is 20 ms. The generated output gating pulses (S_11_–S_55_) from the FSM are grouped according to their dedicated H-bridge circuit (H-Bridge 1 to H-Bridge 5) connections. In the simulation results, samples of output gating pulses for H-Bridge 1 and H-Bridge 5 are shown. The examples of dead times between upper and lower switches (S_2X_ and S_4X_) for H-Bridge 1 (T_DH1_24_) and H-Bridge 5 (T_DH5_24_) are also shown in Figure 16a. The dead-time durations for H-Bridge 1 (T_DH1_24_) and H-Bridge 5 (T_DH5_24_) are 247 µs and 6465 µs, respectively. The duration of dead time for H-Bridge 1 is shorter than that for H-Bridge 5 because the pulse-width duration for H-Bridge 1 is longer than that for H-Bridge 5.

Figure 16b shows the zoomed view of marked area A, which indicates the details of FSM states that generate the output gating pulses (S_11_–S_55_) for the 21-level inverter. The FSM generates the output gating pulse patterns according to state number, defined step-time (t_1_–t_11_) duration for each state, and the internal polarity signal, as illustrated in Figure 8 and listed in Table 4 and Table 5. As mentioned earlier, each state number represents the step-time duration (t_1_–t_11_). Therefore, states St2 to St11 generate the output gating pulses (S_11_–S_55_) according to the step-time duration of t_2_ to t_11_ under Side 1, while states St10 to St1 generate output gating pulses (S_11_–S_55_) for step time t_10_ to t_1_ under Side 0. Similar states of execution are repeated for both positive and negative cycles.

**Figure 17:**
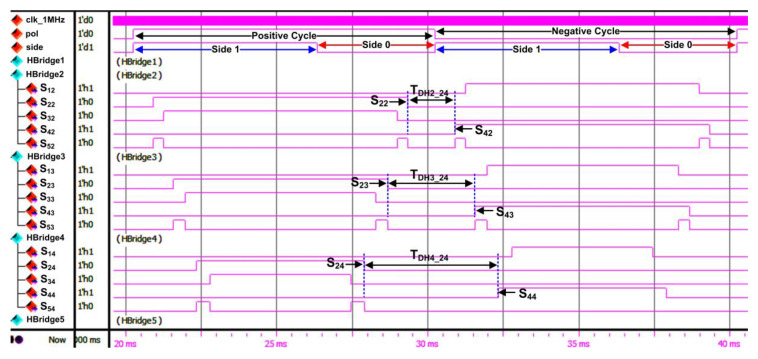
RTL simulation results of the SHE digital switching controller’s output gating pulses for circuits of H-Bridge 2 (S_12_–S_52_), H-Bridge 3 (S_13_–S_53_), and H-Bridge 4 (S_14_–S_54_).

Figure 17 depicts the output gating pulses for circuits of H-Bridge 2 (S_12_–S_52_), H-Bridge 3 (S_13_–S_53_), and H-Bridge 4 (S_14_–S_54_). The pattern of output gating pulses (S_11_–S_55_) and the pulse-width duration obtained from these simulation results were used as references to compare with the real-time hardware measurements for functional verification purposes. The examples of dead times between upper and lower switches (S_2X_ and S_4X_) for H-Bridge 2 (T_DH2_24_), H-Bridge 3 (T_DH3_24_), and H-Bridge 4 (T_DH4_24_) are also shown in Figure 17. The duration of dead time for H-Bridge 2 (T_DH2_24_), H-Bridge 3 (T_DH3_24_), and H-Bridge 4 (T_DH4_24_) is 1583 µs, 2889 µs, and 4445 µs, respectively. It is observed that the dead-time duration increases with the increment of H-bridge number because the pulse-width duration of the switching gating signals decreases as the H-bridge circuit number increases. From the simulation results for all H-bridge circuits, dead-time durations were found to be above the calculated minimum dead-time value required by the IGBT (IKP10N60T) [44] and optocoupler (HCPL-3020) [45] components, as shown in Equation (1). Therefore, the IGBT (IKP10N60T) switches in the H-bridge circuits are safe for operation in the 21-level multilevel inverter.

#### 4.2.2. RTL Simulation Results of SPWM Digital Switching Controller

**Figure 18:**
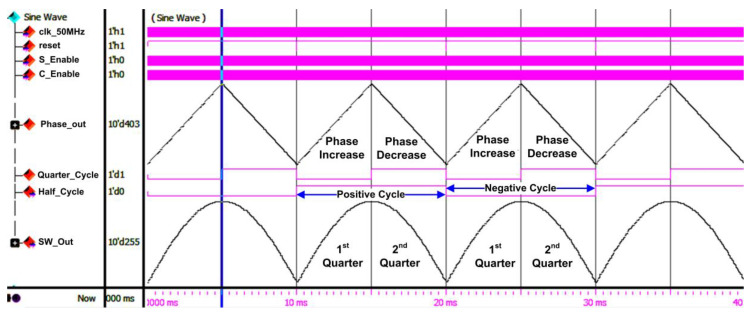
RTL simulation results for the reference sine-wave generation of the SPWM digital switching controller.

Figure 18 depicts the SPWM digital switching controller’s RTL simulation result for reference sine-wave generation (“SW_Out [9:0]”). The “Half_Cycle” signal is used as a reference to generate the positive and negative cycles of the output gating pulses. The duration for each polarity (positive or negative) cycle is 10 ms. Therefore, the total duration to generate the reference stepped sine-wave output waveform is 20 ms. As shown in the simulation result, the phase values (“Phase_Out [9:0]”) are incremented when the “Quarter_Cycle” is at logic LOW (1st Quarter) and decremented when the “Quarter_Cycle” signal is at logic HIGH (2nd Quarter). The phase values (“Phase_Out [9:0]”) are used by the iterative CORDIC sub-module to generate the reference sine-wave signal (“SW_Out [9:0]”).

**Figure 19:**
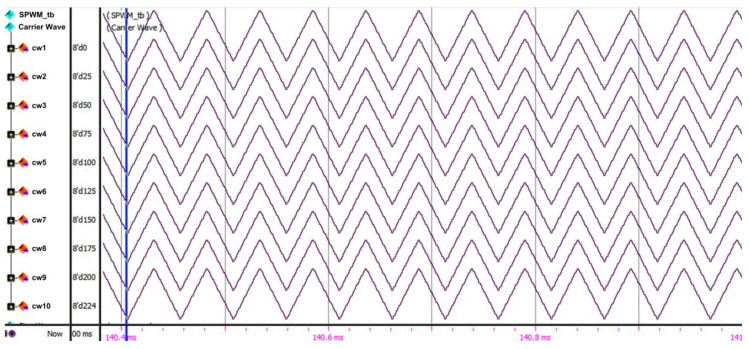
RTL simulation result for the ten carrier-wave (cw1 to cw10) generations of the SPWM digital switching controller.

Figure 19 depicts the RTL simulation result for the generation of ten carrier waves (cw1 to cw10). As depicted in the simulation result, the carrier waves are spaced among one another with an amplitude difference of 25, except for cw10, which has an amplitude difference of 24. This is to ensure that the final maximum peak for cw10 is 255, which is similar to the reference sine-wave maximum-amplitude level.

**Figure 20:**
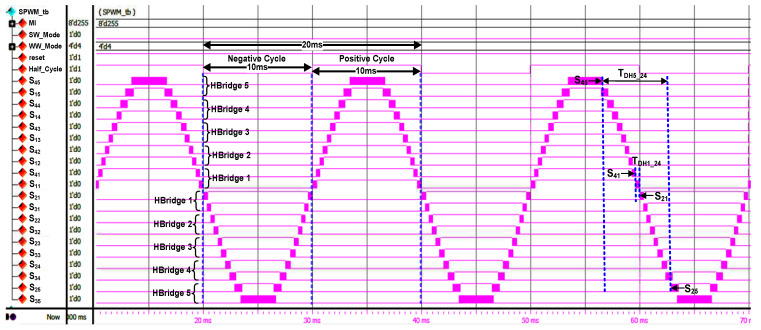
RTL simulation result for the output gating pulses (“S_45_” to “S_35_”) of the SPWM digital switching controller.

Figure 20 depicts the RTL simulation result of the generation of output gating pulses (“S_45_” to “S_35_”), which were connected to the 20 IGBT switches in the H-bridge circuit models for the final 21-level stepped sine-wave generation. As indicated in the simulation result, the “Half-Cycle” signal is used as a reference to generate the output gating pulses for the positive and negative cycles. As shown in Figure 20, the output gating pulses of “S_45_” to “S_11_” are used for positive cycle switching, while the output gating pulses of “S_21_” to “S_35_” are used for negative cycle switching. The patterns of these output gating pulses (“S_45_” to “S_35_”) from the simulation results were used as references to compare with the real-time hardware measurement for functional verification purposes.

The examples of dead times between upper and lower switches (S_2X_ and S_4X_) for H-Bridge 1 (T_DH1_24_) and H-Bridge 5 (T_DH5_24_) are also shown in Figure 20. The measured dead times for H-Bridge 1 (T_DH1_24_) and H-Bridge 5 (T_DH5_24_) are 412 µs and 6253 µs, respectively. It is observed that the dead-time duration increases because the pulse-width duration of the switching gating signals decreases as the H-bridge circuit number increases. From the simulation results for all H-bridge circuits, dead-time durations were found to be above the calculated minimum dead-time value required by the IGBT (IKP10N60T) [44] and optocoupler (HCPL-3020) [45] components, as shown in Equation (1). Therefore, the IGBT (IKP10N60T) switches in the H-bridge circuits are safe for operation in the 21-level multilevel inverter.

### 4.3. Hardware Measurement of SHE and SPWM Digital Switching Controllers

The functionalities of the designed SHE and SPWM digital switching controllers were further verified in real time by downloading the design HDL codes into the Intel FPGA (DE2-115) board to measure the generated output gating pulses from both designs using a digital oscilloscope.

#### 4.3.1. Hardware-Measurement Results of SHE Digital Switching Controller

**Figure 21:**
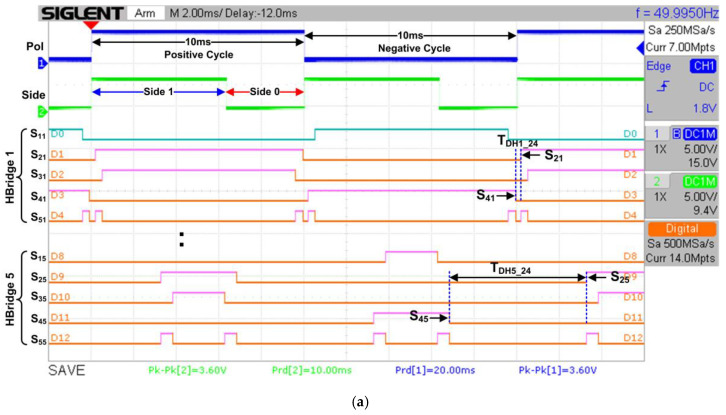

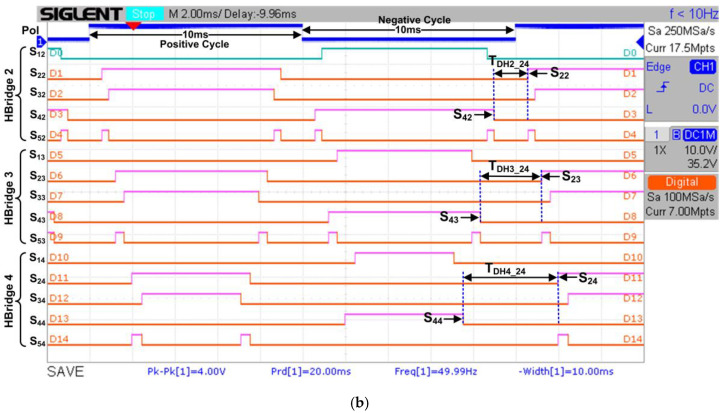
Hardware measurement of the SHE digital switching controller’s output gating pulses: (**a**) Output gating pulses for H-Bridge 1 (“S_11_” to “S_51_”) and H-Bridge 5 (“S_15_” to “S_55_”). (**b**) Output gating pulses for H-Bridge 2 (“S_12_” to “S_52_”), H-Bridge 3 (“S_13_” to “S_53_”), and H-Bridge 4 (“S_14_” to “S_54_”).

Figure 21a depicts the measured output gating pulses for H-Bridge 1 (“S_11_” to “S_51_”) and H-Bridge 5 (“S_15_” to “S_55_”) using a digital oscilloscope. The polarity (“Pol”) and side (“Side”) signals were also measured to indicate the positive and negative cycles, as well as the duration of each cycle (10 ms), which is equivalent to a 20-ms period or 50 Hz frequency. Based on real-time hardware-measurement results, the observed duration and patterns of output gating pulses for H-Bridge 1 and H-Bridge 5 are similar to those obtained in the functional simulation, as shown in Figure 16a. The output gating pulses for H-Bridge 2 (“S_12_” to “S_52_”), H-Bridge 3 (“S_13_” to “S_53_”), and H-Bridge 4 (“S_14_” to “S_54_”) circuits were also measured to validate the output patterns, as depicted in Figure 21b. It is observed that the duration and output patterns for H-Bridge 2 (“S_12_” to “S_52_”), H-Bridge 3 (“S_13_” to “S_53_”), and H-Bridge 4 (“S_14_” to “S_54_”) are also similar to those obtained in the functional simulation, as shown in Figure 17. The examples of dead times between upper and lower switches (S_2X_ and S_4X_) for H-Bridge 1 (T_DH1_24_), H-Bridge 2 (T_DH2_24_), H-Bridge 3 (T_DH3_24_), H-Bridge 4 (T_DH4_24_), and H-Bridge 5 (T_DH5_24_) are also shown in Figure 21. The measured dead-time durations for all H-bridge circuits were found to be similar to those obtained in the simulation, as shown in Figure 16 and Figure 17. These hardware-measurement results validate the design functionality of the SHE digital switching controller in real time.

#### 4.3.2. Hardware-Measurement Results of SPWM Digital Switching Controller

**Figure 22:**
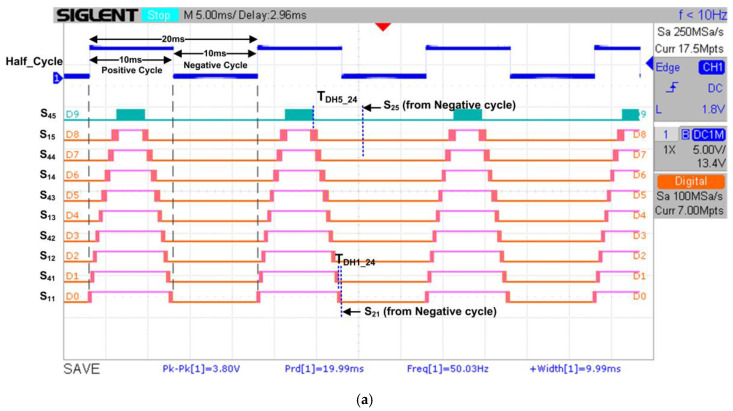

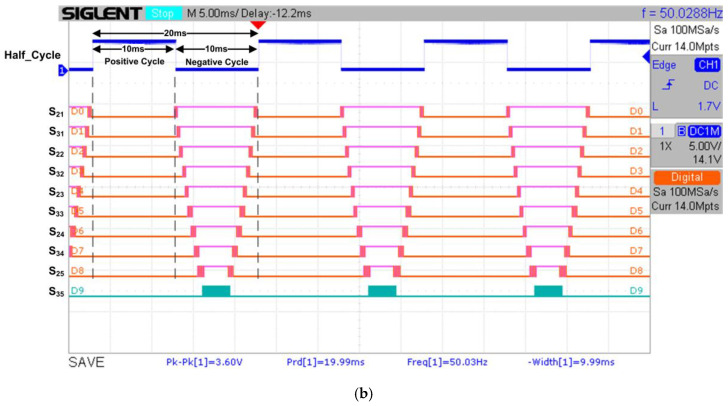
Hardware measurement of the SPWM digital switching controller’s output gating pulses: (**a**) Output gating pulses for positive cycle (“S_45_” to “S_11_”). (**b**) Output gating pulses for negative cycle (“S_21_” to “S_35_”).

Figure 22 depicts the measured output gating pulses (“S_45_” to “S_35_”) of the SPWM digital switching controller for the positive cycle (Figure 22a) and the negative cycle (Figure 22b) using a digital oscilloscope. From the measured results, it is observed that the “Half_Cycle” duration is 20 ms, with 10 ms for positive and negative cycles, respectively. The duration of 20 ms for the “Half_Cycle” signal indicates the period of the stepped sine-output waveform generated once the output gating pulses are connected to the IGBT switches. As depicted in Figure 22a, the output gating pulses for “S_45_” to “S_11_” are activated only during the positive cycle. In contrast, the output gating pulses for “S_21_” to “S_35_” are activated only during the negative cycle, as indicated in Figure 22b. The duration and patterns of these real-time output gating pulses for “S_45_” to “S_35_” were observed to match exactly with those obtained in the functional simulation, as shown in Figure 20. The examples of dead times between upper and lower switches (S_2X_ and S_4X_) for H-Bridge 1 (T_DH1_24_) and H-Bridge 5 (T_DH5_24_) are also shown in Figure 22a. The measured dead-time durations for all H-bridge circuits were found to be similar to those obtained in the simulation, as shown in Figure 20. Therefore, these hardware-measurement results validate the design functionality of the SPWM digital switching controller in real time.

### 4.4. System-Level Verification for 21-Level Multilevel Inverter (HDL Co-Simulation)

This section discusses system-level verification of the simulation results of the designed SHE and SPWM digital switching controllers for the 21-level cascaded H-Bridge multilevel inverter model in the MATLAB Simulink environment. Both the SHE and SPWM digital switching controllers were connected to the IGBT switches, which reside in the five cascaded H-bridge circuits, to observe the stepped sine-wave output waveforms for the 21-level multilevel inverter. The performance of the designed switching controllers was analysed in terms of design functionality in producing the final stepped sine-wave output waveform, the timing duration, and the THD percentage.

#### 4.4.1. System-Level Verification of 21-Level SHE Digital Switching Controller

**Figure 23:**
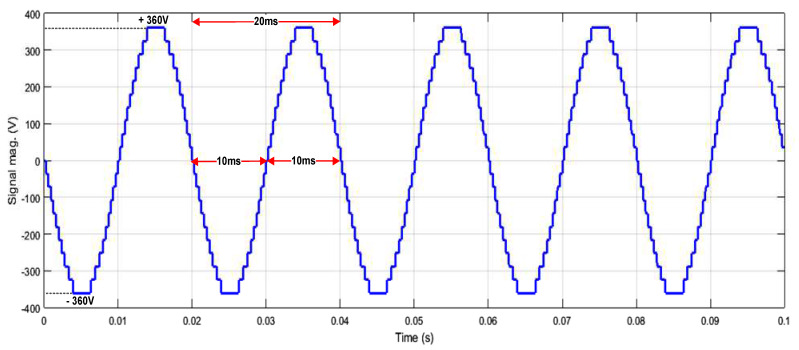
Continuous stepped sine-wave output voltage of the 21-level multilevel inverter using the SHE digital switching controller.

**Figure 24:**
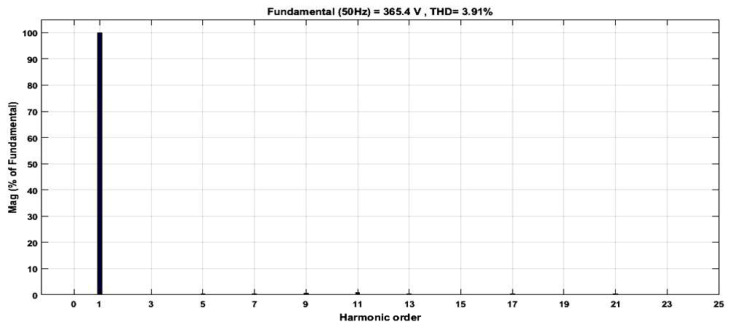
Percentage of THD for the 21-level multilevel inverter using the SHE digital switching controller.

Figure 23 depicts the HDL co-simulation results of the designed SHE digital switching controller (in HDL) connected to 25 IGBT switches in the MATLAB Simulink environment. It is observed that the output gating pulses from the designed SHE digital switching controller were able to generate a 21-level stepped sine-wave output waveform when applied to the five cascaded H-bridge circuit models, with a maximum peak-to-peak voltage of approximately ±360 V_PP_. The duration of the positive and negative cycles of the generated stepped sine-output waveform is 10 ms each. Therefore, the total duration for one complete cycle of the stepped sine-wave output waveform is 20 ms, which is equivalent to 50 Hz. The percentage of THD for this SHE 21-level inverter is 3.91%, as depicted in Figure 24.

#### 4.4.2. System-Level Verification of 21-Level SPWM Digital Switching Controller

**Figure 25:**
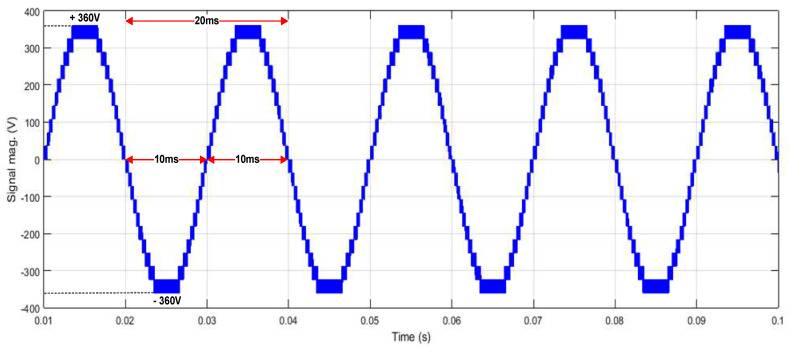
Continuous stepped sine-wave output voltage of a 21-level multilevel inverter using the SPWM digital switching controller with a carrier frequency of 20 kHz.

**Figure 26:**
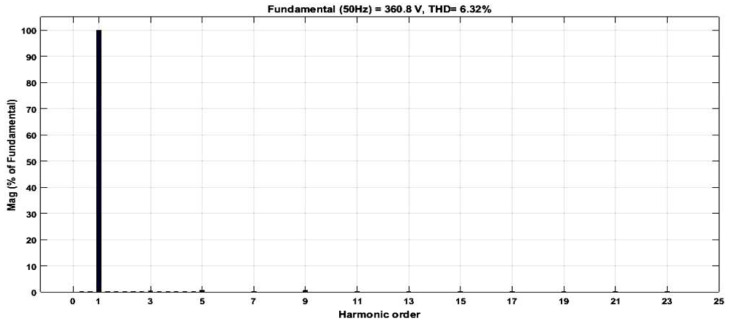
Percentage of THD for the 21-level multilevel inverter using the SPWM digital switching controller with a carrier frequency of 20 kHz.

Figure 25 depicts the HDL co-simulation results of the designed SPWM digital switching controller (in HDL) connected to 20 IGBT switches in the MATLAB Simulink environment. It is observed that the output gating pulses from the designed SPWM digital switching controller were able to generate a 21-level stepped sine-wave output waveform when applied to the five cascaded H-bridge circuit models, with a maximum peak-to-peak voltage of approximately ±360 V_PP_. The duration of the positive and negative cycles of the generated stepped sine-wave output waveform is 10 ms each. Therefore, the total duration for one complete cycle of the stepped sine-wave output waveform is 20 ms, which is equivalent to 50 Hz. The percentage of THD for this SPWM 21-level inverter with a carrier wave frequency of 20 kHz is 6.32%, as depicted in Figure 26.

The THD percentage of the 21-level multilevel inverter using SPWM digital switching was further analysed using several ranges of carrier-wave frequencies, as listed in Table 10. Since the SPWM digital switching controller was able to generate carrier frequencies up to 800 kHz, the SPWM multilevel inverter system was tested with different carrier frequencies, ranging from 1 kHz to 800 kHz. From the listed THD in Table 10, it is observed that the percentage of THD decreases as the carrier-wave frequency increases, when compared with the initial carrier frequency set at 1 kHz. At the typical carrier frequencies of 20 kHz for IGBT switches, the percentage of THD reduces by 7%, and at the typical maximum carrier frequencies of 40 kHz for IGBT switches, the percentage of THD reduces by 9%. At the highest carrier frequency of 800 kHz, the percentage of THD is reduced by 30%, with a THD percentage of 4.73%.

#### 4.4.3. Comparative Analyses of THD

The percentage of THD for both SHE and SPWM digital switching controllers was further compared, as listed in Table 11. The THD for the SPWM digital switching controller was referred to at a carrier frequency of 40 kHz, which is the typical maximum frequency of the IGBT switches. From the obtained results, it is observed that the percentage of THD for the 21-level SHE is 37% less than that of the 21-level SPWM. The higher THD percentage of SPWM compared to SHE is expected due to its high switching frequency, which produces ripples at the output gating pulses and contributes to higher harmonic content. A low-pass filter circuit can be used at the output of a 21-level SPWM circuit to remove the harmonic content at a higher frequency range, which can result in a lower THD value.

The THD percentages of the 21-level multilevel inverter using the SHE and SPWM digital switching controllers as modulation techniques were further compared with other research work. Table 12 compares the THD of the 21-level multilevel inverter for the SHE digital switching controller to the THD reported in other research studies that used low-switching-frequency modulation techniques. It is observed that most of the THD percentages obtained from other research work are greater than 4%. The lowest THD percentage comes from Khasim et al. (2021), at only 3.49%, obtained by using the asymmetric DC method [23]. The THD percentage of our designed SHE digital switching controller is the second lowest, at 3.91%, compared to others. For this 21-level multilevel inverter, only a few research papers have presented a design architecture of the switching controller in hardware. Although Khasim et al. (2021) reported the lowest THD percentage, the switching modulation was implemented using the dSPACE RIT1104 controller equipment, which is quite bulky in size and costly [23]. Niraimathi and Seyezhai (2020) implemented three types of switching-controller algorithms in a microcontroller (PIC16F877A), and the obtained THD percentages are greater than those obtained with our designed SHE switching controller. Bhavani and Manoharan (2021) implemented the switching controller in FPGA-Spartan 6 SP601, but no design architectures were explained in the published work [11], and the obtained THD percentage is 47% greater than that obtained with our designed SHE controller. By comparing the THD percentage with that reported in other research work, it can be concluded that our designed SHE digital switching controller not only provides a simple design architecture with minimal hardware logic resources but also has a low THD percentage.

The THD percentage of the 21-level multilevel inverter using the SPWM digital switching controller as the modulation technique was also compared with that reported in other research work. Table 13 compares the THD of the 21-level multilevel inverter for the SPWM digital switching controllers to that reported in other research studies that used a multicarrier with high switching frequency (F_SW_) for the SPWM modulation technique.

It is observed that the THD of our designed SPWM digital switching controller is 58% and 41% lower than the THD obtained by Agrawal and Bansal (2017) [1] and Hema Latha et al. (2018) [7], respectively. However, the THD of our designed SPWM digital switching controller is 9% and 18% greater than the THD obtained by Singh et al. (2018) [52] and Mahato et al. (2018) [53], respectively. Since our SPWM digital switching controller was designed to handle higher switching frequencies of up to 800 kHz, where MOSFET power switches can be used to operate at higher-frequency values, a lower THD percentage could also be obtained. Although Salgado et al. (2013) employed FPGA—Spartan 3E as a design platform, the THD percentage for the 21-level multilevel inverter was not stated in the published work. Additionally, the digital switching controller designed by Salgado et al. (2013) required a large amount of memory (RAM) storage for the 500 sampled points of sine-wave data [18].

## 5. Conclusions

The design and implementation of SHE and SPWM digital switching controllers using the Intel FPGA (DE2-115) board for 21-level multilevel inverter applications has been thoroughly expounded. Both digital switching controllers were designed with minimal hardware complexity and logic resources. For the SHE digital switching controller, the design only requires a four-bit FSM and a 13-bit counter to generate the 21-level stepped sine-wave output waveform. For the SPWM digital switching controller, the design employed an iterative CORDIC algorithm to generate the reference sinusoidal waveform at 50 Hz and a six-bit up-down counter with nine adders to generate ten carrier-wave signals. The CORDIC algorithm requires only a small amount of LUT to store eight arctan angles besides adder, subtractor, and shifter logic circuits. Therefore, the proposed SHE and SPWM digital switching controllers are much simpler in terms of design architecture and do not consume significant hardware logic resources compared to digital switching controllers reported in other research work, which require the usage of large amounts of BRAM to store the sampled-points data as well as complex softcore processor architectures.

The detailed internal design architecture of SHE and SPWM digital switching controllers, their operation in digital hardware logic, and their overall design implementation flow have been illustrated and elucidated. The functional behaviour of the SHE and SPWM digital switching controllers was tested using Intel Quartus II Modelsim software. The designed controllers were also synthesised and downloaded to the Intel FPGA (DE2-115) board for hardware measurement and real-time verification.

The synthesised logic-gate results indicate that total logic elements and registers for the SPWM digital switching controller (369/114,480) are almost double that of the SHE digital switching controller (186/114,480). However, the total utilisation of logic elements for each of the digital switching controllers (SHE or SPWM) was still low (less than 1%) compared to other designs. The maximum frequency (F_max_) of the SHE digital switching controller was 6% greater than that of the SPWM digital controller when a 50 MHz clock was used as a reference. The execution speed of the SHE digital switching controller implemented in the FPGA (Cyclone IV E) chip was found to be 95.39% faster compared to that implemented in the microcontroller (PIC16F877A). The execution speed of the SHE digital switching controller was further increased to a maximum of 99.97% faster than that of the microcontroller (PIC16F877A) when the maximum clock frequency was used (F_max_).

From the RTL simulations and hardware-measurement results, it can be concluded that the designed SHE and SPWM digital switching controllers function correctly, conforming to the functional behaviour of output gating pulse patterns with accurate timing duration. The measured dead-time delays from the RTL simulations and hardware-measurement results for all H-bridge circuits were found to be greater than the minimum dead-time-delay (3.05 µs) requirement of the IGBT (IKP10N60T) and optocoupler (HCPL-3020) components.

The system-level performance of the designed switching controllers was verified using HDL co-simulation in the MATLAB Simulink environment. The designed SHE and SPWM digital switching controllers were able to produce a 21-level stepped sine-wave output waveform with ±360 V_pp_ and a frequency of 50 Hz. The THD percentage of the 21-level SHE digital switching controller (3.91%) was found to be 37% less than that of the SPWM digital switching controller (6.17%). The THD percentages of SHE and SPWM were also compared with those obtained in other research work. The findings from the comparative analyses indicate that the designed SHE and SPWM switching controllers were not only able to provide simplified design architectures with minimum logic resources but also an acceptably lower THD percentage.

In conclusion, the proposed SHE and SPWM digital switching controllers have been proven to have minimal hardware complexity in terms of design architecture and require few hardware logic resources (less than 1% of LEs utilisation) when synthesised in an FPGA (Cyclone IV E) chip. Both switching controllers were able to function correctly when tested on a real-time FPGA (DE2-115) board and produced a safe dead-time delay for both upper and lower IGBT switches in all H-bridge circuits. The execution speed of the SHE digital switching controller was improved by a maximum of 99.97% when compared with that of the microcontroller (PIC16F877A). Furthermore, both switching controllers produced the desired 21-level stepped sinusoidal output waveform (with ±360 V_pp_) with a low THD percentage when tested in HDL co-simulation in MATLAB Simulink. These promising results are very beneficial for the development of portable power converters in solar PV applications that demand small digital-controller devices with low power consumption.

## Figures and Tables

**Table 1:** Active power switches for a 21-level multilevel inverter for SHE modulation technique.

Active Switches	Output Voltage (V)
S_11_, S_41_, S_12_, S_42_, S_13_, S_43_, S_14_, S_44_, S_15_, S_45_	+5.0 V_DC_
S_11_, S_41_, S_12_, S_42_, S_13_, S_43_, S_14_, S_44_, S_45_, S_55_	+4.5 V_DC_
S_11_, S_41_, S_12_, S_42_, S_13_, S_43_, S_14_, S_44_	+4.0 V_DC_
S_11_, S_41_, S_12_, S_42_, S_13_, S_43_, S_44_, S_45_	+3.5 V_DC_
S_11_, S_41_, S_12_, S_42_, S_13_, S_43_	+3.0 V_DC_
S_11_, S_41_, S_12_, S_42_, S_43_, S_53_	+2.5 V_DC_
S_11_, S_41_, S_12_, S_42_	+2.0 V_DC_
S_11_, S_41_, S_42_, S_52_	+1.5 V_DC_
S_11_, S_41_	+1.0 V_DC_
S_41_, S_51_	+0.5 V_DC_
All power switches are inactive	+0.0 V_DC_
S_21_, S_51_	−0.5 V_DC_
S_21_, S_31_	−1.0 V_DC_
S_21_, S_31_, S_22_, S_52_	−1.5 V_DC_
S_21_, S_31_, S_22_, S_32_	−2.0 V_DC_
S_21_, S_31_, S_22_, S_32_, S_23_, S_53_	−2.5 V_DC_
S_21_, S_31_, S_22_, S_32_, S_23_, S_33_	−3.0 V_DC_
S_21_, S_31_, S_22_, S_32_, S_23_, S_33_, S_24_, S_54_	−3.5 V_DC_
S_21_, S_31_, S_22_, S_32_, S_23_, S_33_, S_24_, S_34_	−4.0 V_DC_
S_21_, S_31_, S_22_, S_32_, S_23_, S_33_, S_24_, S_34_, S_25_, S_55_	−4.5 V_DC_
S_21_, S_31_, S_22_, S_32_, S_23_, S_33_, S_24_, S_34_, S_25_, S_35_	−5.0 V_DC_

**Table 2:** IGBT switch arrangement for each H-bridge circuit for 21-Level SHE and SPWM multilevel inverters.

H-Bridge X	SHE Digital Switching Controller (S_1X_ to S_5X_)	SPWM Digital Switching Controller (S_1X_ to S_4X_)
H-Bridge 1	S_11_, S_21_, S_31,_ S_41_,S_51_	S_11_, S_21_, S_31_, S_41_
H-Bridge 2	S_12_, S_22_, S_32,_ S_42_, S_52_	S_12_, S_22_, S_32_, S_42_
H-Bridge 3	S_13_, S_23_, S_33,_ S_43_, S_53_	S_13_, S_23_, S_33_, S_43_
H-Bridge 4	S_14_, S_24_, S_34,_ S_44_, S_54_	S_14_, S_24_, S_34_, S_44_
H-Bridge 5	S_15_, S_25_, S_35,_ S_45_, S_55_	S_15_, S_25_, S_35_, S_45_

**Table 3:** Conversion of optimised switching angles (θ_1_–θ_10_) to switching times (T_1_–T_10_).

Optimised Switching Angles (θ)	Switching Times, (µs)
θ_1_ = 2.16	T_1_ = 120
θ_2_ = 8.26	T_2_ = 459
θ_3_ = 14.24	T_3_ = 791
θ_4_ = 20.23	T_4_ = 1124
θ_5_ = 26.00	T_5_ = 1444
θ_6_ = 33.00	T_6_ = 1833
θ_7_ = 40.00	T_7_ = 2222
θ_8_ = 48.00	T_8_ = 2667
θ_9_ = 58.18	T_9_ = 3232
θ_10_ = 68.02	T_10_ = 3779

**Table 4:** Calculation of step-time duration (t_1_–t_11_).

Level	Step Time, (t)	Calculation	Duration, (µs)
0.0 V_DC_	t_1_	2T_1_	240
±0.5 V_DC_	t_2_	T_2_ − T_1_	339
±1.0 V_DC_	t_3_	T_3_ − T_2_	332
±1.5 V_DC_	t_4_	T_4_ − T_3_	333
±2.0 V_DC_	t_5_	T_5_ − T_4_	321
±2.5 V_DC_	t_6_	T_6_ − T_5_	389
±3.0 V_DC_	t_7_	T_7_ − T_6_	389
±3.5 V_DC_	t_8_	T_8_ − T_7_	444
±4.0 V_DC_	t_9_	T_9_ − T_8_	566
±4.5 V_DC_	t_10_	T_10_ − T_9_	547
±5.0 V_DC_	t_11_	(5 ms − T_10_) × 2	2442

**Table 5:** Output gating patterns of the SHE digital switching controller (S_11_–S_55_) for each state.

Present States	Polarity (pos = 1, neg = 0)	Output (S_11_–S_55_)
St1 (±0.0 V_DC_)	1 0	00000_00000_00000_00000_00000 00000_00000_00000_00000_00000
St2 (±0.5 V_DC_)	1 0	00011_00000_00000_00000_00000 01001_00000_00000_00000_00000
St3 (±1.0 V_DC_)	1 0	10010_00000_00000_00000_00000 01100_00000_00000_00000_00000
St4 (±1.5 V_DC_)	1 0	10010_00011_00000_00000_00000 01100_01001_00000_00000_00000
St5 (±2.0 V_DC_)	1 0	10010_10010_00000_00000_00000 01100_01100_00000_00000_00000
St6 (±2.5 V_DC_)	1 0	10010_10010_00011_00000_00000 01100_01100_01001_00000_00000
St7 (±3.0 V_DC_)	1 0	10010_10010_10010_00000_00000 01100_01100_01100_00000_00000
St8 (±3.5 V_DC_)	1 0	10010_10010_10010_00010_00000 01100_01100_01100_01001_00000
St9 (±4.0 V_DC_)	1 0	10010_10010_10010_10010_00000 01100_01100_01100_01100_00000
St10 (±4.5 V_DC_)	1 0	10010_10010_10010_10010_00011 01100_01100_01100_01100_01001
St11 (±5.0 V_DC_)	1 0	10010_10010_10010_10010_10010 01100_01100_01100_01100_01100

**Table 6:** Amplitude-level disposition for ten carrier waves.

Carrier Waves	Amplitude	Triangular Amplitude (Start–Max–End)
cw1	cw1 = cw1 + 1	0–31–0
cw2	cw2 = cw1 + 25	25–56–25
cw3	cw3 = cw1 + 50	50–81–50
cw4	cw4 = cw1 + 75	75–106–75
cw5	cw5 = cw1 + 100	100–131–100
cw6	cw6 = cw1 + 125	125–156–125
cw7	cw7 = cw1 + 150	150–181–150
cw8	cw8 = cw1 + 175	175–206–175
cw9	cw9 = cw1 + 200	200–231–200
cw10	cw10 = cw1 + 224	224–255–224

**Table 7:** Synthesis summary report of SHE and SPWM digital switching controllers using the Intel field-programmable gate array (FPGA) (Cyclone IV E) chip.

Items	SHE Digital Switching Controller	SPWM Digital Switching Controller
Family name	Cyclone IV E	Cyclone IV E
Device	EP4CE115F29C7	EP4CE115F29C7
Total logic elements	186/114,480 (<1%)	369/114,480 (<1%)
Total registers	59	108
Total pins	29/529 (5%)	36/529 (7%)
Total memory bits	0/3,981,312 (0%)	0/3,981,312 (0%)
Embedded multiplier 9-bit elements	0/532 (0%)	1/532 (<1%)
Total PLLs	0/4 (4%)	0/4 (0%)
F_max_ (slow 1200 mV 85 °C) (Clock reference = 50 MHz)	198.06 MHz	152.25 MHz

**Table 8:** 21-level SHE digital switching controller implemented in FPGA (Cyclone IV E) and microcontroller (PIC16F877A).

SHE Digital Switching Controller	Operating Frequency	State/Machine Cycle	Execution Time (s)
FPGA (Cyclone IV E)	FSM clock frequency = 1 MHz	40 states	40 µs
	F_MAX_ = 137.99 MHz (Clock reference = 1 MHz)	40 states	0.289 µs
Microcontroller (PIC16F877A)	F_OSC_ = 4 MHz	868 machine cycles	868 µs

**Table 9:** Comparison of design architectures of digital switching controllers for SHE and SPWM modulation techniques.

Proposed by	Modulation Technique	Hardware Logic Design Method	FPGA Device Synthesised Logic Gate
Juarez Abad et al. (2021) [19]	SPWM (7-level)	Sine wave: 1024 × 32 ROM Carrier wave: 1000 × 32-bit BRAM	Xilinx Spartan 6-SLX45 LCs: 2464 (5.6%) Slices: 385 (5.6%) BRAM: 14 (12%)
Sarker et al. (2021) [20]	HD-SPWM (3-level)	Sine wave: BRAM (Fs = 4 MHz) Carrier wave: digital pulse	Xilinx Spartan 3-3S400 LCs: 378 (4.6%) Slices: 168 (4.6%) BRAM: 1 (1%)
Sarker et al. (2020) [2]	SPWM (9-level)	Sine wave: BRAM (256 samples) Carrier wave: 4 up-down counters	Xilinx Spartan 6-SLX9 LCs: 9152 (100%) Slices: 1430 (100%)
Nikhil et al. (2018) [42]	SPWM (NA)	Sine wave: softcore 32-bit RISC TSK-3000A and SRAM. Carrier wave: up-down counter	Xilinx Spartan 3AN NA
Atoui et al. (2018) [17]	SPWM (5-level)	Sine wave: ROM (2000 samples) Carrier wave: up-down counter	Xilinx Spartan 6-SLX45 LCs: 1126 (2.6%) Slice LUTs: 703 (2.6%)
Ranganathan et al. (2016) [27]	SPWM (NA)	Sine wave: 7 BRAM Softcore 32-bit RISC Microblaze	Xilinx Spartan 6-SLX4 LCs: 2110 (55%) BRAM: 4 (58%)
Khalil et al. (2020) [5]	SHE (3-level)	PWM: timers Shared and distributed memories Dual softcore Microblaze	Xilinx Spartan 6 NA
Halim et al. (2014, 2015 and 2017) [12,13,14]	SHE (7, 13 and 9-level)	Sine wave: LUT (5000 samples) and combinational logic gates	Intel Cyclone II LEs: 1173 (4%) Combo Func: 1170 (4%) Register: 53
Our Work	SPWM (21-level)	Sine wave: LUT (8 arctan angles) Carrier wave: one 6-bit up-down counter and 9 adders.	Intel Cyclone IV E LEs: 369 (<1%) Register: 108
Our Work	SHE (21-level)	Sine wave: 4-bit FSM and 13-bit counter	Intel Cyclone IV E LEs: 186 (<1%) Register: 59

**Table 10:** Total harmonic distortion (THD) of the 21-level inverter for the SPWM digital switching controller at different carrier-wave frequencies (1 kHz to 800 kHz).

Carrier Wave	THD %	% THD Reduction from Carrier Wave = 1 kHz
1 kHz	6.79	0
3 kHz	6.43	5
5 kHz	6.41	6
10 kHz	6.36	6
20 kHz	6.32	7
40 kHz	6.17	9
80 kHz	5.94	13
100 kHz	5.82	14
200 kHz	5.35	21
400 kHz	5.08	25
800 kHz	4.73	30

**Table 11:** THD comparison of 21-level inverters for SHE and SPWM digital switching controllers.

Digital Switching Controller	Level	THD %
SHE	21	3.91
SPWM (cw = 40 kHz)	21	6.17

**Table 12:** Comparison of THD of a 21-level multilevel inverter using a low-switching-frequency modulation technique.

Proposed by	Modulation Technique (Low Switching Frequency)	Hardware	Software	THD % (Simulation)
Bhavani and Manoharan (2021) [11]	GWO Algorithm	FPGA—Spartan 6	Matlab	7.41
Hema Latha and Banakara (2018) [7]	-	-	Matlab	10.67
Islam et al. (2018) [50]	Equal-Phase Method Half-Height Method	-	Matlab	13.3 4.09
Niraimathi and Seyezhai (2020) [51]	GA Algorithm PSO Algorithm MGWO-PI-PWM Algorithm	Microcontroller (PIC16F877A)	Matlab	4.5 7.2 6.51
Khasim et al. (2021) [23]	-	dSPACE RIT1104 Controller	Matlab	3.49
Our work (SHE)	MhyPSO Algorithm	FPGA—DE2 115	Matlab	3.91

**Table 13:** Comparison of THD of 21-level multilevel inverter using SPWM modulation technique.

Proposed by	Modulation Strategy (High Switching Frequency)	Hardware	Software	THD % (Simulation)
Agrawal and Bansal (2018) [1]	Asymmetrical SPWM (PD) (F_SW_ = 2 kHz)	-	Matlab	14.52
Singh et al. (2016) [52]	SPWM (APOD) (F_SW_ = 5 kHz)	-	Matlab	5.61
Hema Latha and Banakara (2018) [7]	SPWM (F_SW_ = 3 kHz)	-	Matlab	10.45
Mahato et al. (2019) [53]	Asymmetrical SPWM (PD) (F_SW_ = 3 kHz)	-	Matlab	5.08
Salgado et al. (2013) [18]	SPWM (F_SW_ = 3 kHz)	FPGA—Spartan 3E	-	-
Our work (SPWM)	SPWM (PD-PWM) (F_SW_ = 40 kHz)	FPGA—DE2 115	Matlab	6.17

## Data Availability

Not applicable.
